# Prediction of dynamic allostery for the transmembrane domain of the sweet taste receptor subunit, TAS1R3

**DOI:** 10.1038/s42003-023-04705-5

**Published:** 2023-04-03

**Authors:** Keisuke Sanematsu, Masato Yamamoto, Yuki Nagasato, Yuko Kawabata, Yu Watanabe, Shusuke Iwata, Shingo Takai, Kiyoshi Toko, Toshiro Matsui, Naohisa Wada, Noriatsu Shigemura

**Affiliations:** 1 Section of Oral Neuroscience, Graduate School of Dental Science, Kyushu University, 3-1-1 Maidashi, Higashi-ku, Fukuoka, 812-8582 Japan; 2 Oral Health/Brain Health/Total Health Research Center, Graduate School of Dental Science, Kyushu University, 3-1-1 Maidashi, Higashi-ku, Fukuoka, 812-8582 Japan; 3 Research and Development Center for Five-Sense Devices, Kyushu University, 744 Motooka, Nishi-ku, Fukuoka, 819-0395 Japan; 4 Department of General Dentistry, Division of Interdisciplinary Dentistry, Faculty of Dental Science, Kyushu University, 3-1-1 Maidashi, Higashi-ku, Fukuoka, 812-8582 Japan; 5 Department of Bioresources and Biosciences, Faculty of Agriculture, Graduate School of Kyushu University, 744 Motooka, Nishi-ku, Fukuoka, 819-0395 Japan; 6 Institute for Advanced Study, Kyushu University, 744 Motooka, Nishi-ku, Fukuoka, 819-0395 Japan

**Keywords:** G protein-coupled receptors, Neurophysiology

## Abstract

The sweet taste receptor plays an essential role as an energy sensor by detecting carbohydrates. However, the dynamic mechanisms of receptor activation remain unclear. Here, we describe the interactions between the transmembrane domain of the G protein-coupled sweet receptor subunit, TAS1R3, and allosteric modulators. Molecular dynamics simulations reproduced species-specific sensitivity to ligands. We found that a human-specific sweetener, cyclamate, interacted with the mouse receptor as a negative allosteric modulator. Agonist-induced allostery during receptor activation was found to destabilize the intracellular part of the receptor, which potentially interfaces with the Gα subunit, through ionic lock opening. A common human variant (R757C) of the TAS1R3 exhibited a reduced response to sweet taste, in support of our predictions. Furthermore, histidine residues in the binding site acted as pH-sensitive microswitches to modulate the sensitivity to saccharin. This study provides important insights that may facilitate the prediction of dynamic activation mechanisms for other G protein-coupled receptors.

## Abstract (web summary)

Molecular dynamics simulations and functional assays of the transmembrane domain of the sweet taste receptor subunit, TAS1R3 reveal mechanisms on the allostery of sweet receptor activation or inactivation and pH-dependent sensitivity to saccharin.

## Introduction

**Fig. 1:**
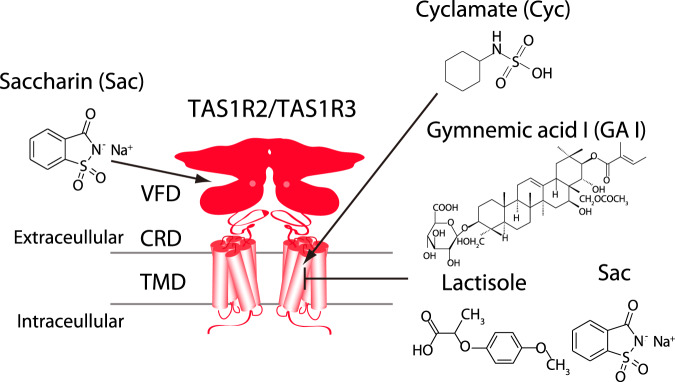
Interaction site for sweet substances and modulators. The human taste type 1 receptor member 2 (hTAS1R2; left) and hTAS1R3 (right) are shown schematically together with the basic molecular structures of sweet substances and inhibitors. Saccharin (Sac) binds to the Venus flytrap domain (VFD) of TAS1R2 as an agonist. Cyclamate (Cyc) acts as a positive allosteric modulator in the transmembrane domain (TMD) of hTAS1R3. Gymnemic acid I (GAI), Sac and lactisole interact with the TMD of hTAS1R3 as negative allosteric modulators. CRD cysteine-rich domain.

Sweet taste receptors act as energy sensors by detecting carbohydrates, which are a source of calories. Prediction of the mechanisms of sweet taste receptor activation would facilitate the development of taste modifiers for patients with diabetes mellitus and obesity. Taste type 1 receptor (TAS1R) family members are expressed in taste receptor cells and are class C G protein-coupled receptors (GPCRs). The heterodimeric sweet taste receptor formed by TAS1R2 and TAS1R3 recognizes diverse sweet-tasting substances such as sugars, sweet amino acids, artificial sweeteners and sweet proteins^1,2^. The TAS1R subunits consist of three principal domains (Fig. 1). The large extracellular amino-terminal domain contains a bilobed Venus flytrap domain (VFD) that is responsible for the binding of sweet compounds at an orthosteric site. A cysteine-rich domain (CRD) transmits the movement of the VFD to the heptahelical transmembrane domain (TMD), which interacts with a G protein for signal transduction in the cytoplasm^3,4^. The TAS1R2/TAS1R3 heterodimer has multiple binding domains, and the TMD serves as an allosteric modulator site^5–8^ (Fig. 1 and Supplementary Fig. 1). Cyclamate is an artificial sweetener that interacts with the TMD of human TAS1R3 (hTAS1R3) as a positive allosteric modulator (PAM), whereas gymnemic acids (triterpene glycosides derived from the plant *Gymnema sylvestre*) and lactisole bind to the same domain and act as negative allosteric modulators (NAMs) to inhibit sweet taste^5,7^. Saccharin, another artificial sweetener, acts not only as an agonist of the hTAS1R2 through binding to its VFD but also (at high concentrations) as a NAM of the hTAS1R3 through an interaction with its TMD^9,10^. The binding of these compounds to the TMD of the TAS1R3 is species-specific and differs between human receptors and mouse receptors.

Upon activation or inactivation of a receptor, allostery is considered to mediate long-range communication between distant receptor sites through inter- and intra-protein networks of coupled residues^11^. The dimeric architecture of class C GPCRs is essential for the transduction of agonist binding at the VFDs into the activation of G proteins, which interact with the TMDs. A previous study of the crystal structures of the VFD of the medaka fish Tas1r2/Tas1r3 receptor demonstrated that the binding of amino acids leads to closure of the VFDs accompanied by dimer rearrangement^12^. Although the molecular structure of the TAS1R3 TMD has not been solved, the closure of the VFD is presumed to cause a conformational change in the downstream TMD that may interact with the heterotrimeric G protein for signal transduction. Therefore, elucidation of the dynamics of the TMD would improve our understanding of how allosteric conformational changes in the overall structure of the TAS1R2/TAS1R3 lead to activation or inactivation of the receptor following the binding of a ligand.

Previous studies of the sweet taste receptor have identified steric binding sites using functional assays based on heterologous expression systems and docking simulations based on homology modeling of the TMD^5–8,13–15^. The overall dynamic mechanisms of receptor activation have been investigated using molecular dynamics (MD) simulations of a full-length heterodimer^16,17^. However, the detailed mechanisms by which allosteric modulators induce activation or inactivation through allosteric interactions involving the TMD remain unknown.

Our previous study of the sweet-modifying effect of miraculin showed that acidification promoted receptor activation due to an interaction between the protonated sweet taste receptor and protonated miraculin^18^. Our findings raised the possibility that a pH-dependent alteration in the charges of residues might act as a microswitch that modifies the affinity of the sweet taste receptor to its ligands.

Here, we provide a MD-based explanation of the species-specific sensitivity of the TAS1R3 TMD to ligands and describe the allosteric mechanisms that operate following the binding of a PAM or NAM. We also show that protonation of histidine residues in the TMD acts as a microswitch that potentially changes the sensitivity of the TMD to saccharin.

## Results

### MD simulations predict the species-specific sensitivity of the TAS1R3 TMD to ligands

**Fig. 2:**
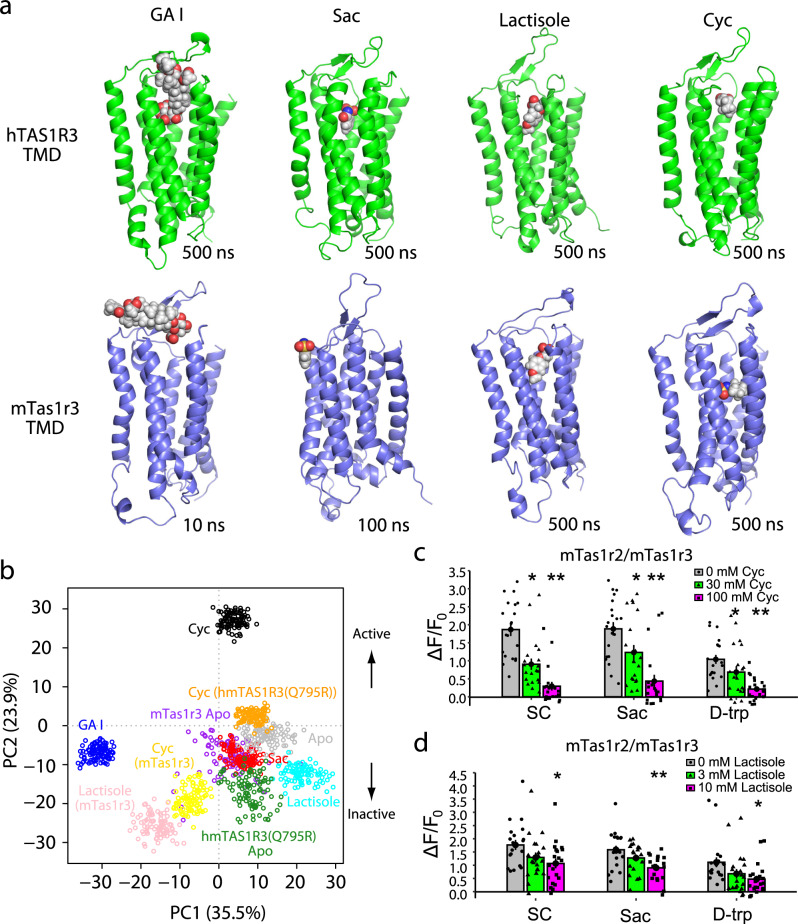
Species-specific dynamics of the transmembrane domain (TMD) of taste type 1 receptor member 3 (TAS1R3). **a** Snapshots of the TMD of human (upper, green) and mouse (lower, blue) TAS1R3 docked with allosteric modulators. The snapshots were obtained at the indicated times during molecular dynamics (MD) simulations. The TAS1R3 TMD and allosteric modulators are displayed as ribbons and spheres colored by atom type, respectively. Gymnemic acid (GAI) and saccharin (Sac) moved to the outside of the mouse receptor. **b** Principal component analysis (PCA) of the MD trajectories (the last 100 ns) using instantaneous conformations. Each point represents a structure, and the structures were color-coded as indicated below. The human TAS1R3 (hTAS1R3) TMD complexed with cyclamate (Cyc, black) and the hmTAS1R3(Q795R) TMD complexed with Cyc (a cyclamate-activated variant, orange) represent active GPCR structures. The hTAS1R3 TMDs complexed with GAI (blue), Sac (red) and lactisole (cyan) represent inactive GPCR structures. Also shown are the mTas1r3 TMD complexed with lactisole (pink), mTas1r3 TMD complexed with Cyc (yellow), apo form of the hTAS1R3 TMD (gray), apo form of the hmTAS1R3(Q795R) TMD (forest green) and apo form of the mTas1r3 TMD (purple). **c**, **d** The mouse sweet receptor heterodimer (mTas1r2/mTas1r3) was expressed in HEK293 cells along with a Gα subunit (G16-gust44). Calcium mobilization was measured in response to each of the following sweeteners in the absence or presence of Cyc or lactisole: SC45647 (0.3 mM), saccharin (10 mM) and D-tryptophan (10 mM). Cyc (**c**) and lactisole (**d**) fully or partially inhibited the responses to all the sweeteners tested. Data are expressed as the mean ± S.E. of 20 cells (*n* = 3). *P < 0.05 vs. control, **P < 0.01 vs. control (one-way ANOVA and Tukey’s post-hoc test).

Since the molecular structure of the TAS1R3 TMD has not yet been solved, we constructed a homology model using the TMD of the metabotropic glutamate receptor subtype 1 (mGluR1) in complex with a NAM (4-fluoro-N-methyl-N-{4-[6-(propan-2-ylamino)pyrimidin-4-yl]-1,3-thiazol-2-yl}benzamide) as a template (PDB ID: 4OR2)^19^. After running docking simulations, we introduced the receptor-compound complexes into a lipid bilayer with ionized water and performed MD simulations. First, we examined whether the species-specific sensitivity to ligands was reproducible for the TMDs of human and mouse Tas1r3 (mTas1r3) docked with allosteric modulators (Fig. 2). MD simulations lasting 500 ns demonstrated that gymnemic acid I, saccharin, lactisole and cyclamate stably resided within the putative binding pocket of hTAS1R3 TMD, and the stabilities of the MD systems were confirmed by root-mean-square displacement calculations (Fig. 2a, Supplementary Figs. 2–6 and Supplementary Movie 1). In the mouse receptor model, gymnemic acid I and saccharin moved to the outside of the binding pocket of the receptor, whereas lactisole and cyclamate remained bound to the receptor (Fig. 2a, Supplementary Figs. 7, 8 and Supplementary Movie 2). Next, we employed principal component analysis (PCA) to examine the relationships between different geometrical conformations of the hTAS1R3 Cα segment in complex with cyclamate, saccharin, gymnemic acid I or lactisole throughout the MD trajectories (Fig. 2b and Supplementary Fig. 9). The results showed that the distribution of hTAS1R3 complexed with cyclamate and the distributions of hTAS1R3 complexed with NAMs were located on opposite sides of the PC2 axis, which possibly determines whether or not these conformations are active. After projection of other trajectories onto the axes of the largest principal components, the mouse TMD models with cyclamate or lactisole were located on the same side of hTAS1R3 complexed with NAMs. As a control of the active model, distribution of a cyclamate-activated variant, hmTAS1R3(Q795R) complexed with cyclamate (a chimera in which the sequence of the human receptor from transmembrane helix (TM) helix VI (position 752) is replaced with that of the mouse receptor with a humanized variant Q795R)^6^ was located on the same side of hTAS1R3 complexed with cyclamate compared to the apo forms of hmTAS1R3(Q795R). The above results suggest that the mouse models complexed with cyclamate or lactisole represented inactive states. To confirm these predictions, we performed a sweet taste receptor assay using a heterologous expression system (Fig. 2c, d, Supplementary Fig. 10 and Supplementary Table 1). High concentrations of cyclamate and lactisole significantly inhibited the responses to SC45647, saccharin and D-tryptophan in HEK293 cells expressing the mouse sweet taste receptor, indicating that lactisole and cyclamate interact with the mouse sweet taste receptor as NAMs (*Ps* < 0.05, one-way ANOVA and Tukey’s post-hoc test). Thus, our simulation demonstrates that there are species-specific interactions between allosteric modulators and the TAS1R3 TMD.

### Microswitches in the TMD of TAS1R3 drive activation or inactivation

**Fig. 3:**
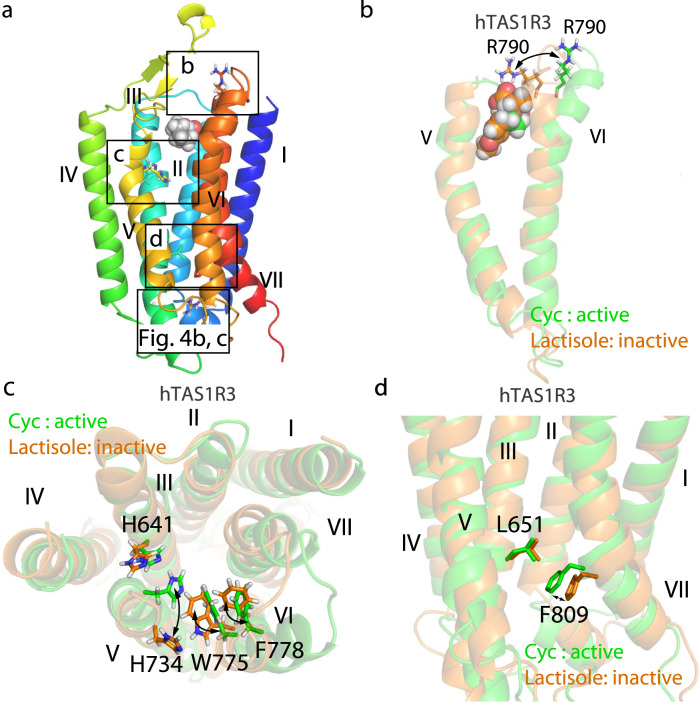
Microswitches in the transmembrane domain (TMD) of taste type 1 receptor member 3 (TAS1R3). **a** Overall view of the TMD of TAS1R3. The boxes represent the regions indicated in the subsequent panels. **b** Superimposition of transmembrane helices (TM) V and VI of the human TAS1R3 (hTAS1R3) bound with cyclamate (Cyc;) (active, green) and lactisole (inactive, orange). **c** Superimposition of His-641^6.40^, His-734^5.44^, Trp-775^6.50^ and Phe-778^6.53^ of the Cyc-bound (active, green) and lactisole-bound (inactive, orange) hTAS1R3. **d** Superimposition of L651^3.50^ and F809^7.47^ of the hTAS1R3 bound with Cyc (active, green) and lactisole (inactive, orange).

**Fig. 4:**
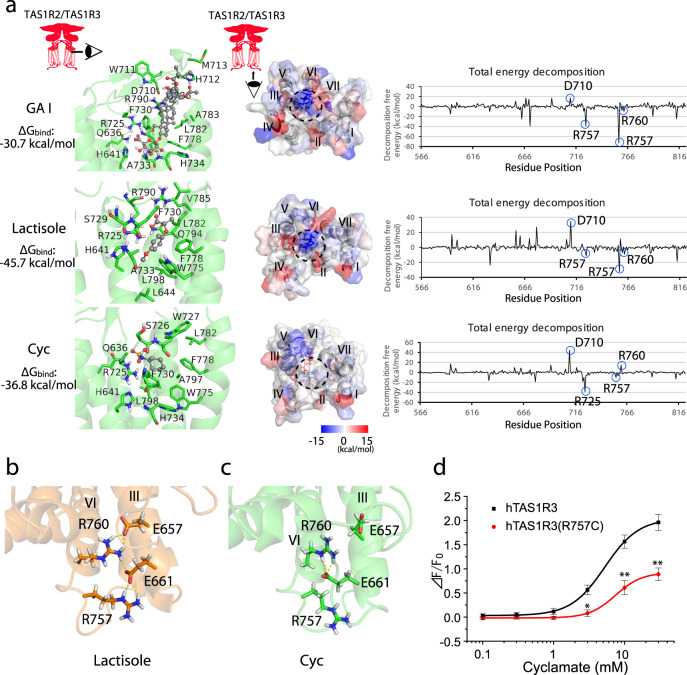
Allostery of the transmembrane domain (TMD) of the human taste type 1 receptor member 3 (hTAS1R3) leads to conformational changes at the Gα subunit interface. **a** Left, detailed views of the TAS1R3 TMD binding pocket with gymnemic acid (GAI), lactisole or cyclamate (Cyc) after 500 ns of molecular dynamics simulation. The binding free energies (ΔG_bind_) determined by the molecular mechanics/Poisson–Boltzmann surface area method are indicated. Middle, molecular surface of the intracellular region of the TAS1R3 TMD colored according to decomposition energy. The dotted line indicates the predicted interface with the Gα subunit. Right, total energy decomposition for each residue of the hTAS1R3. **b** Preservation of the salt bridges between Glu-657^3.56^ in transmembrane helix (TM) III and Arg-760^6.35^ in TM VI of hTAS1R3 bound with lactisole (500 ns). **c** Breakage of the salt bridges between Glu-657^3.56^ in TM III and Arg-760^6.35^ in TM VI of hTAS1R3 bound with Cyc. Yellow dotted lines indicate hydrogen bonds (500 ns). **d** HEK293 cells were transiently transfected with hTAS1R3 or hTAS1R3(R757C) along with hTAS1R2 and Gα16-gust44. The responses of the receptors to various concentrations of cyclamate were examined. Data are expressed as the mean ± S.E. of 16–19 cells (*n* = 3). *P < 0.05, **P < 0.01 for the comparison between hTAS1R3 and hTAS1R3(R757C) (two-way ANOVA and post-hoc *t*-test).

Next, we examined the contribution of each residue to the first two principal components (Supplementary Fig. 11). Differential comparisons between PC1 and PC2 showed that the regions determining the active or inactive conformational state were mainly the binding sites on TM III, TM V and TM VI as well as an intracellular region of TM VI that potentially interfaces with the G protein alpha subunit^20–24^ (Supplementary Fig. 11). Superimposition of the lactisole- and cyclamate-bound models showed that cyclamate induced the outward movement of the extracellular parts of TM VI and VII when compared with lactisole (Fig. 3a–c). Arg-790^7.28^ formed a salt bridge with a carboxy group of lactisole but not with cyclamate (Fig. 4a and Supplementary Fig. 5). The cyclamate-bound model exhibited contacts between His-641^3.40^ in TM III and His-734^5.44^ in TM V, whereas NAMs did not induce any contacts between these residues (Fig. 3c and Supplementary Fig. 12). Trp-775^6.50^ and Phe-778^6.53^ in TM VI were rotated clockwise in the cyclamate-bound hTAS1R3 TMD in comparison to their counterparts in the lactisole-bound model, which is in accordance with the movement of His-734^5.44^ (Fig. 3c). In the central part of the hTAS1R3, the distance between Leu-651^3.50^ in TM III and Phe-809^7.47^ in TM VII was shorter for cyclamate than for lactisole (Fig. 3d and Supplementary Fig. 12). In the functional assay, the mutation of Leu-651^3.50^ with alanine abolished the sensitivity to sweet compounds, whereas the mutation did not affect the cell surface expression (Supplementary Figs. 13 and 14). The results indicate that Leu-651^3.50^ is important for the receptor function.

### Allosteric modulators induce conformational changes in the TMD

Next, we calculated the binding free energy (ΔG_bind_) of these compounds using the molecular mechanics/Poisson–Boltzmann surface area (MM/PBSA) method^25^. Energy decomposition was performed to estimate which residues contributed to a stable binding structure (Fig. 4a). As a result, Arg-725^ex2^ and Asp-710^ex2^ were identified as common stabilized and destabilized residues in the binding of gymnemic acid, lactisole, and cyclamate, respectively. Arg-725^ex2^ at the end of the extracellular loop 2 is a key residue for the bindings with these compounds (Supplementary Figs. 3, 5 and 6). The destabilization of Asp-710^ex2^ needs to be re-evaluated through full-length receptor analysis, as Asp-710^ex2^ in the middle of extracellular loop 2 may interact with the CRD. The projection of energy decomposition to molecular surface showed that the intracellular interface of the hTAS1R3 TMD was stabilized by the binding of a NAM but not by the binding of cyclamate. These results indicate that stabilization of the intracellular interface by a NAM may lead to a reduced potential interaction with the Gα subunit. The inactive state of rhodopsin, a class A GPCR, is stabilized by the formation of an ionic lock between Arg^3.50^ in the DRY motif of TM III and Asp^6.33^ in the intracellular region of TM VI^26–32^. Our MD simulations demonstrated the formation of salt bridges between Glu-657^3.56^ in TM III and Arg-760^6.35^ in TM VI of the TAS1R3 TMD when it was complexed with lactisole, saccharin or gymnemic acid I (Fig. 4b, Supplementary Fig. 12 and Supplementary Movie 3), which is similar to the ionic lock in a class A GPCR. By contrast, we found that the allosteric effects induced by cyclamate caused breakage of the salt bridges between these residues (Fig. 4c, Supplementary Fig. 12 and Supplementary Movie 4).　The cyclamate-bound hmTAS1R3(Q975R) also exhibited broken salt bridges between Glu-657^3.56^ and Arg-765^6.35^, which are equivalent to Arg-760^6.35^ in the hTAS1R3 (Supplementary Fig. 12). This finding suggests that ionic lock opening helps to drive the intermediate state from the inactive state to the active state. Each mutation of Glu-657^3.56^ and Arg-760^6.35^did not reduce the surface expression but abolished the sensitivity to sweet compounds, suggesting these residues plays key roles in the receptor activity (*F*4,10 = 1.31, *P* > 0.05 for ratio, *F*4,95 = 0.13, *P* > 0.05 for intensity, one-way ANOVA; Supplementary Fig. 13, 14 and Supplementary Table 2). Energy decomposition analysis indicated that Arg-757^6.32^, which lies next to Arg-760^6.35^ on the helical pitch, stabilized the receptor-NAM complex (Fig. 4a). The above result suggests that Arg-757^6.32^ may destabilize the intracellular region of the apo receptor, facilitating transition to the active state following agonist binding. Therefore, we examined the functional effects of a single nucleotide polymorphism (R757C) in the hTAS1R3. The maximal responses to cyclamate, D-tryptophen, and neohesperidin dihydrochalcone (NHDC) were significantly lower for the minor allele variant, hTAS1R3(R757C), than for hTAS1R3 possessing the major allele Arg-757^6.32^ (*F*1,165 = 19.05, *P* < 0.001 for cyclamate, *F*1,128 = 7.35, *P* < 0.05 for cyclamate, *F*1,155 = 10.07, *P* < 0.001 for cyclamate, two-way ANOVA, effect of genotype; Fig. 4d, Supplementary Fig. 15 and Supplementary Table 3), whereas there was no significant difference in surface expression (*F*4,10 = 0.064, *P* > 0.05 for ratio, *F*4,95 = 0.70, *P* > 0.05 for intensity, one-way ANOVA; Supplementary Table 2). These results strongly support our hypothesis that destabilization induced by Arg-757^6.32^ in the intracellular region enhances the efficacy of receptor activation by an agonist.

### Histidine residues act as pH-sensitive microswitches to modify the binding affinity of saccharin

**Fig. 5:**
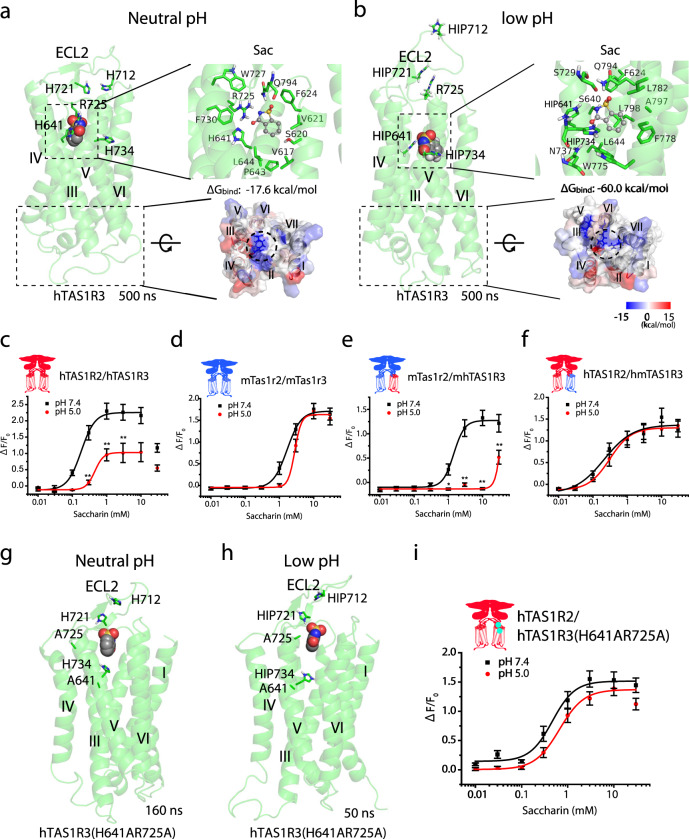
Protonation of histidine residues modifies the binding affinity of saccharin. Saccharin was stably located in the binding pocket of human taste type 1 receptor member 3 (hTAS1R3) after a 500 ns molecular dynamics simulation at neutral pH (**a**) and at low pH (**b**). Left, overall view of the TAS1R3 bound with saccharin (Sac). Upper right, detailed view of the binding pocket of the Sac-bound TAS1R3 and the binding free energy (ΔG_bind_). Lower right, the molecular surface of the intracellular region of the Sac-bound TAS1R3 colored according to decomposition energy. Protonated histidine residues are indicated as HIP. ECL, extracellular loop. **c**–**f** The transmembrane domain (TMD) of hTAS1R3 mediates the pH-dependence of the sensitivity to Sac. HEK293 cells were transiently transfected with full-length and/or chimeric TAS1R2 and TAS1R3 as well as Gα16-gust44. The TAS1R2 (left) and TAS1R3 (right) are shown schematically. The full-length and chimeric TAS1Rs are labeled as human (h, red) or mouse (m, blue), with hm denoting the chimera containing the VFD and CRD of the human receptor coupled to the TMD of the mouse receptor, and mh indicating the chimera comprising the VFD and CRD of the mouse receptor coupled to the TMD of the human receptor. The responses of the receptors to different concentrations of Sac were examined at neutral pH (7.4) and at low pH (5.0). **g**, **h** Sac moved to the outside of the binding pocket of the hTAS1R3 variant at neutral or low pH during MD simulations. Overall views of the Sac-bound TAS1R3(H641AR725A) variant are shown at neutral pH (**g**) and low pH (**h**). **i** Mutation of hTAS1R3 attenuated the pH-dependence of the sensitivity to Sac. HEK293 cells were transiently transfected with TAS1R3 or TAS1R3(H641AR725A) together with TAS1R2 and Gα16-gust44. The responses of the receptors to different concentrations of Sac were examined at neutral pH (7.4) and low pH (5.0). Data are expressed as the mean ± S.E. of 15–20 cells (*n* = 3). *P < 0.05, **P < 0.01 for the comparison between neutral pH and low pH (two-way ANOVA and post-hoc *t*-test).

Our previous study showed that the affinity of the sweet taste receptor for its ligands could potentially vary depending on the charges of its residues, which acted as pH-sensitive microswitches^18^. To examine this possibility, we focused on four histidine residues (His-641^6.40^, His-712^ex2^, His-721^ex2^ and His-734^5.44^) located on the extracellular region of the hTAS1R3 TMD, which is a potentially pH-sensitive site. Our MD study indicated that saccharin was able to bind strongly to the TMD of human TAS1R3 (Fig. 5a, b and Supplementary Fig. 4, 16). Calculation of the binding free energy indicated that binding was further stabilized when these histidine residues were protonated (Fig. 5a, b and Supplementary Fig. 17, 18). Among these four residues, His-641^6.40^ and His-734^5.44^ were found to interact with saccharin in the binding site. To test this prediction, we performed a sweet taste receptor assay using a heterologous expression system. The results showed that HEK293 cells expressing hTAS1R2/hTAS1R3 exhibited significantly reduced responses to saccharin under low pH conditions (*F*_1,294_ = 10.33, *P* < 0.01, two-way ANOVA, effect of pH; Fig. 5c and Supplementary Table 4). This effect was not observed in HEK293 cells expressing the mouse receptor (*F*_1,266_ = 0.07, *P* > 0.05, two-way ANOVA, effect of pH; Fig. 5d and Supplementary Table 4). Low pH also significantly suppressed the response to saccharin in a human-mouse chimeric receptor (mTas1r2/mhTAS1R3) created by replacing the TMD of the mouse receptor with that of the hTAS1R3 (*F*_1,294_ = 27.86, *P* < 0.001, two-way ANOVA, effect of pH; Fig. 5e and Supplementary Table 4). By contrast, pH exerted no effects on saccharin responses in a chimeric receptor (hTAS1R2/hmTAS1R3) made by replacing the TMD of the human receptor with that of the mTas1r3 (*F*_1,217_ = 0.004, *P* > 0.05, two-way ANOVA, the effect of pH; Fig. 5f and Supplementary Table 4). The above findings indicate that the suppressing effect of low pH on saccharin responses was mediated by the TMD of the hTAS1R3 but not the TMD of the mTas1r3. Next, we performed mutational analyses using MD simulations and the sweet taste receptor assay. Previous studies had shown that the replacement of His-734 with alanine^5.44^ produces a receptor with low responsiveness^5,6^. Therefore, we evaluated the effects of replacing both His-641^6.40^ and Arg-725^ex2^, a positively charged residue thought to be important for binding, with alanine. MD simulations predicted that saccharin moved to the outside of the binding pocket of this variant under neutral or low pH conditions (Fig. 5g, h), indicating a weaker interaction. Furthermore, the sweet taste receptor assay revealed that the double mutation of the hTAS1R3 (H641AR725A) attenuated the pH-dependence of its sensitivity to saccharin, although the single mutations of His-641or Arg-725^ex2^, and the mutation of Gln-636^3.35^, involved in saccharin binding, did not completely reduce the pH-dependence. (*F*_1,236_ = 2.5, *P* > 0.05, two-way ANOVA, effect of pH; Fig. 5i, Supplementary Fig. 19 and Supplementary Table 4). These results suggest that a pH-sensitive site is present in the TMD of the hTAS1R3.

## Discussion

The present study has demonstrated that the species-specific sensitivity of the sweet taste receptor to allosteric modulators could be reproduced in an in silico experimental system. In addition to the effects of species-specific residues at the binding site (Supplementary Figs. 3–8), this difference in sensitivity could be affected by allostery involving species-specific residues at distant positions in the receptor. Previous studies based on docking simulations were focused mainly on the steric binding between the sweet taste receptor and its ligands^5–8,13–15^, hence these studies were unable to demonstrate whether binding induced receptor activation. By using MD simulations to compare the predicted structures of receptors bound to a PAM or NAM, we succeeded in predicting whether the structures of other ligand-receptor complexes reflected the process of activation or inactivation. We found that cyclamate, which activates human sweet taste receptors, was unable to activate mouse sweet taste receptors but instead bound to the pocket and acted as a NAM. In addition, high concentrations of lactisole (3–10 mM) exerted a weak inhibitory effect on the mouse sweet taste receptor due to a decrease in binding affinity, whereas a much lower concentration of lactisole (0.3 mM) was found to completely inhibit the response of the human sweet taste receptor^7^. Our simulation potentially could be used to predict allosteric mechanisms and the ability of a ligand to activate a receptor in other GPCRs. Furthermore, our study provides important insights that may facilitate the future development of new artificial sweeteners and taste modifiers.

Our simulations identified specific residues participating in binding with saccharin, lactisole, and cyclamate (Supplementary Figs. 4–6). In saccharin binding, Arg-725^ex2^, Gln-636^3.35^, and Gln-794^7.32^ interact with the negatively charged group of saccharin by a salt bridge or hydrogen bonds. Single mutation of Arg-725^ex2^ and Gln-636^3.35^ did not affect the pH-dependence of saccharin sensitivity, suggesting that other residues involved in binding may complement the effect of the mutation. Mutations of Gln-794^7.32^ have been reported to abolish the receptor activation, so mutation-induced changes in binding affinity to saccharin could not be followed. The double mutant of H641A and R725A reduced the pH-dependence of saccharin sensitivity, suggesting that these amino acid residues may be involved in binding with saccharin. In the interaction with lactisole, Arg-790^7.28^, Arg-725^ex2^, and Phe-778^6.53^ form salt bridges or π-πinteraction with the negatively charged carboxy group and phenoxy ring of lactisole, respectively. Although the Arg-790^7.28^ mutation does not affect lactisole sensitivity so much, mutations of Arg-725^ex2^ and Phe-778^6.53^ are reported to reduce the sensitivity to lactisole^5,7^, supporting our prediction. Cyclamate binds with Arg-725^ex2^, Gln-794^7.32^, and Gln-636^3.35^ through the formation of salt bridges and hydrogen bonds. Gln-636^3.35^ is a key residue for sweet receptor activation induced by cyclamate^6^. Mutation of Arg-725^ex2^ tended to reduce the sensitivity to cyclamate (Supplementary Fig. 20 and Supplementary Table 5, *P* = 0.053, two-way ANOVA, effect of genotype). These results are consistent with our predictions.

Previous studies of molecular structures determined by X-ray crystallography and cryo-electron microscopy reported that microswitches can modulate the active state of a GPCR^24,33,34^. In our simulations, the binding of a PAM induced the outward movement of Arg-790^7.28^ in the extracellular part of TM VII. This movement is considered to help the intracellular region of TM VII shift inwards through the moment, which would be consistent with X-ray structure analyses of class A GPCRs^24^. This movement of TM VII following activation brings Phe-809^7.47^ closer to Leu-651^3.50^ in TM VI. Phe-809^7.47^ and Leu-651^3.50^ are considered equivalent to Tyr^7.53^ in the NPxxY motif of TM VII and Ile^3.46^ in TM III of class A GPCRs, which make contact during activation. From the functional assay, the heterodimer of hTAS1R2/hTAS1R3(L651A) did not constitute a functional receptor, supporting our prediction. In addition, we identified microswitches such as His-734^5.44^, Trp-775^6.50^ and Phe-778^6.53^. The coordination of the counterclockwise movement of His-734^5.44^ in TM V with the clockwise movements of Trp-775^6.50^ and Phe-778^6.53^ in TM VI resulted in outward movements of the extracellular parts of TM VI and TM VII. This shift of TM VI is entirely consistent with a key movement observed in other class C GPCRs such as the calcium-sensing receptor and mGluR2^33,34^. Trp^6.50^ is highly conserved as a toggle switch motif (WxxFxP) in class C GPCRs, similar to the Trp^6.48^ toggle switch in the CWxP motif of class A GPCRs^33–35^. The toggle switch movement in class A GPCRs induces the opening of TM VI at its intracellular end. By contrast, the distinct movement of the toggle switch in class C GPCRs leads to inward rotation of the intracellular half of TM VI.

Activation of class A GPCRs involves the outward movement of the intracellular segments of TM V and VI. In the intracellular portion of a class A GPCR, Arg^3.50^ in the DRY motif of TM III forms an ionic lock with Asp^6.30^ or Glu^6.30^ in TM VI, which stabilizes the inactive state^24,26^. When this bond is broken, the receptor transitions to the active state, which is associated with the outward movement of TM V and VI. This movement is believed to be a key transition that facilitates the interaction of the receptor with the Gα subunit. In this study, we observed that Glu-657^3.56^ in TM III formed an ionic lock with Arg-760^6.35^ in TM VI in the NAM-bound model of the hTAS1R3 TMD, which is reminiscent of the ionic lock between Arg^3.50^ in the DRY motif and Asp^6.30^ or Glu^6.30^ in class A GPCRs. The PAM-bound model exhibited ionic lock opening. Our observations are supported by studies of other class C GPCRs. For example, mGluR1 complexed with a NAM and the apo form of mGluR5 both have an ionic lock between Lys^3.50^ and Glu^6.35^ with opposite charges of hTAS1R3^19^, whereas this ionic lock is broken in a mGluR2 complexed with a PAM and a G protein and in the active form of mGluR5^33,36^. The binding of a NAM is thought to stabilize the intracellular region, block the potential interaction with the Gα subunit and maintain the inactive state. On the other hand, binding of a PAM induces a transition to an intermediate structure in which salt bridges between Arg-760^6.35^ and Glu-657^3.56^ are allosterically broken due to the inward rotation of TM VI, creating an active state that may interact with a G protein. As is the case with other class C GPCRs^33,34^, the PAM-bound hTAS1R3 did not exhibit the large outward opening of the intracellular part of TM VI that is observed in class A GPCRs. In our functional assay, each mutation of Glu-657^3.56^ and Arg-760^6.35^ abolished the sweet receptor activity induced by the different binding sites, including the VFD of hTAS1R2 for SC45647, saccharin, and D-tryptophan, and the TMD of hTAS1R3 for cyclamate. The results indicate the interaction between Glu-657^3.56^ and Arg-760^6.35^ play an important role in the receptor dynamics regardless of the activation pathway.

Our energy decomposition analysis showed that Arg-757^6.32^ is involved in the stabilization of the intracellular region following the binding of a NAM. This residue is thought to destabilize the intracellular region of the apo receptor due to the repulsion between Arg-757^6.32^ and Arg-760^6.35^, which are positively charged, and Arg-757 may help to break the salt bridges between Arg-760^6.35^ and Glu-657^3.56^ upon receptor activation. A common human variant (R757C) with a neutral charge is thought to help stabilize the intracellular region of the apo receptor and make it difficult for the salt bridges between Arg-760^6.35^ and Glu-657^3.56^ to be cleaved. This is consistent with the finding that the hTAS1R2/hTAS1R3(R757C) receptor showed a downward shift in the concentration-response curve to cyclamate, D-tryptophan and another PAM for the TMD of TAS1R3, NHDC. In addition to forming a heterodimer with TAS1R2, TAS1R3 also forms a heterodimer with TAS1R1, which serves as an umami receptor. Our previous study using psychophysiology techniques and a heterologous expression system showed that the R757C variant of hTAS1R3 reduced umami sensitivity^37^, which would be consistent with the effect of this variant to decrease the sensitivity of the sweet taste receptor.

In whole receptor activation of mGluR5, agonist binding at the VFD leads to repositioning the TMDs to contact each other for signal initiation through the interaction between the CRD and the second extracellular loops of the TMD^4^. The sweet receptor activation by binding with sweet compounds at the VFD is also assumed to induce a similar rearrangement of each domain. However, it remains unknown whether PAM and NAM binding to the TMD induce or suppress the rearrangements of each domain. Cyclamate has been shown to act as an enhancer on the umami receptors TAS1R1/TAS1R3, potentially affecting the interactions between each domain^38^. On the other hand, the inhibitory effect of gymnemic acids shows a downward shift in the concentration-response curve, suggesting that it may block the receptor activation pathway. In this study, the R757C mutation causes a downward shift in the concentration-response curve to sweet compounds even in the different binding domains (D-tryptophan at the VFD of hTAS1R2 vs. cyclamate at the TMD of hTAS1R3) and in the different binding forms within the same domain (NHDC vs. cyclamate). The results suggest that Arg-757^6.32^ is important for regulating receptor activation. Further study is required to analyze intramolecular interactions between the other domains of the heterodimer.

For the model, we assumed the intracellular part of the TAS1R3-TMD as the G protein interaction site. However, several independent studies suggest that the G protein preferentially interacts with the TAS1R2-TMD, either with the heterodimeric receptor or when evaluating the functions of monomers^38,39^. Therefore, it should be considered that our predictions are reflected only in the interaction between the TAS1R3-TMD and G proteins.

Our previous studies have reported that the sensitivity of sweet taste receptors may be modulated by pH through protonation of histidine residues. In this study, we focused on histidine residues located in the extracellular part of the hTAS1R3 TMD and showed that the binding affinity of the receptor for saccharin was altered by a reduction in pH. Protonated histidine residues (as found at low pH) were found to interact with the negatively charged group of saccharin, and ΔG_bind_ was lower at acidic pH than at neutral pH, indicating stable binding. Saccharin is known to bind to the VFD of TAS1R2 as an agonist as well as interact with the TMD of TAS1R3 as a NAM. The heterologous expression system experiments performed in the present study revealed that pH-dependent changes in saccharin sensitivity were mediated by the TMD of hTAS1R3. The pH-induced changes in saccharin sensitivity were not due to a direct effect of pH on the action of the saccharin as an agonist at the TAS1R2 but resulted from an enhancement of saccharin’s action as a NAM in the hTAS1R3 TMD. This proposal is supported by the results of the MD simulations, which showed polar interactions among protonated His-641^6.40^, protonated His-734^5.44^ and the negatively charged group of saccharin. Previous studies have reported that mutation of His-734^5.44^ produces receptors with low responsiveness^5,6^. Therefore, we performed MD simulations of a receptor in which His-641^6.40^ and Arg-725^ex2^ (two residues in the binding pocket that would be positively charged at low pH) were mutated to alanine. The results showed that saccharin was released from the binding site under neutral and even acidic conditions, which would predict a lower sensitivity to saccharin. A decrease in the pH-dependence of saccharin sensitivity was also observed in the heterologous expression system, demonstrating that the histidine residue in the binding pocket is involved in the pH-dependence of saccharin sensitivity.

Although a psychophysiological study was not performed at this time, our results suggest that the sweetness of saccharin decreases at lower pH in humans. Saccharin has been shown to inhibit the effects of other sweet compounds acting on TAS1R2-VFD and TAS1R3-TMD^10^, praising a possibility that lower pH may similarly cause a decrease in the sensitivity to other sweet compounds when mixed with saccharin.

In general, when combined with the following amino acid residues of His-641^6.40^ within two helical pitches towards both of extracellular and intracellular sides, histidine residue is conserved at about 20% of human GPCRs (from GPCRdb: https://gpcrdb.org/). It is possible that receptors with a histidine residue at around this position exhibit pH-sensitive ligand binding. If so, the sensitivity of these receptors to their ligands would be altered under acidic conditions such as those associated with cancer and inflammation, which in turn might affect their bioactivity and potentially contribute to disease pathogenesis. Therefore, drug design processes should consider the effects of local environmental pH on ligand sensitivity.

In conclusion, the present study has improved our understanding of the processes underlying activation of the sweet taste receptor. Our research provides important insights that may facilitate MD-based discovery of endogenous ligands and drugs that target GPCRs.

## Methods

### Residue numbering

The general numbering of residues in the TMD of TAS1R3 follows the primary sequence of TAS1R3. Superscripted residue numbers follow the generic numbering system of Ballesteros and Weinstein (for class A GPCRs) and Pin^40,41^ (for class C GPCRs) based on GPCRdb^17^.

### Molecular modeling

Based on the sequence alignment reported by previous studies^5,9,42^, homology models of hTAS1R3 and mTas1r3 were generated by residue replacement using Modeller 9.20^43^ and the mGluR1 as a template (PDB ID 4OR2)^19,40,44^. The structures of gymnemic acid I, lactisole, saccharin and cyclamate (cyclohexyl sulfamate) were obtained from ZINC version 15^19^. The geometry of these compounds was fully optimized by the ab initio quantum chemistry method at the B3LYP/6–31 G(d) level using the Gaussian 16 A.03 package^45^. Gymnemic acid I, lactisole, saccharin, and cyclamate were introduced into the TAS1R3 TMD models using Autodock 4.0^46^. The systems were viewed in the freely available open-source PyMOL molecular visualization system^47^ or Maestro 12.6 (Schrödinger, LLC, New York, NY). Each of the ligand-receptor complexes (∼4140 atoms) was inserted into a pre-equilibrated palmitoyl-oleoyl-phosphatidylcholine (POPC, 64 lipids per leaflet) bilayer using the membrane builder on the CHARMM-GUI web server (www.charmm-gui.org)^48–50^. The restrained ESP (RESP) charges of the ligands were generated by the antechamber program implemented in AMBER18. The AMBER ff14SB^51^, lipid17^52^ and general AMBER force field 2^53^ were used for proteins, lipids and ligands, respectively. Each system was solvated in a TIP3P water model (∼11,950 molecules)^54^ and neutralized with Na^+^/Cl^−^ (150 mM). Total number of atoms were ∼57220. Energy minimization was performed using the steepest descent method for 2500 steps followed by the conjugated gradient method for 2500 steps.

### MD simulations, structural analyses and free energy calculations

We used AMBER 18^55^ for both the equilibration and production processes together with the SHAKE algorithm^56^ based on the protocols provided by the CHARMM-GUI web server. All-atom MD simulations of the 15 systems (hTAS1R3 TMD complexed with saccharin, lactisole, gymnemic acid I or cyclamate, hTAS1R3 TMD (low pH) complexed with saccharin, hTAS1R3(H641AR725A) TMD complexed with saccharin, hTAS1R3(H641AR725A) TMD (low pH) complexed with saccharin, apo form of hTAS1R3 TMD, mTas1r3 TMD complexed with saccharin, lactisole, gymnemic acid I or cyclamate, apo form of mTas1r3, hmTAS1R3(Q795R) complexed with cyclamate, and apo form of hmTAS1R3(Q795R)) were performed under periodic boundary conditions. The short-range cutoff was 9 Å for nonbonded interactions. The long-range electrostatic interactions were calculated by the Particle Mesh Ewald (PME) summation method^57^. The systems were heated up to 310.0 K. During the equilibration run, an NVT (constant particle number, volume and temperature) simulation was performed with a 1-fs time step for 250 ps. Subsequently, an NPT (constant particle number, pressure and temperature) simulation was carried out with a 1-fs time step (for 0.125 ns) and a 2-fs time step (for 1.5 ns). Various positional and dihedral restraint potentials were applied with gradual reduction. The production run was performed with a 2-fs time step for 500 ns without any restraint potential. Each calculation was carried out using ITO in the Research Institute for Information Technology, Kyushu University. The cpptraj module and Bio3d^58^ were used to compute the structural analyses. The equilibrium state of all the simulated models was determined by computing the root-mean-square deviation. The PCA and H-bond calculations for every 100 structures (the last 100 ns) were used to investigate the relevant motions and structural details of the studied complexes, respectively. The trajectories of the apo forms of hTAS1R3, hmTAS1R3(Q795R) (a chimera in which the sequence of the human receptor from TM helix VI (from position 752) is replaced with that of the mouse receptor with a humanized variant Q795R)^6^ and mTas1r3, hmTAS1R3(Q795R) complexed with cyclamate and mTas1r3 complexed with cyclamate or lactisole were projected onto the axes of the largest principal components determined by geometrical conformations of the hTAS1R3 Cα segment in complex with cyclamate, saccharin, gymnemic acid I or lactisole. The MM/PBSA method was employed to calculate the binding free energy (ΔG_bind_) and predict the binding site, essential amino acid residues for ligand binding, and binding affinity^25,59^.

### Preparation of expression constructs

Human and mouse TAS1Rs, their chimeras, and Gαl6-gust44 constructs were inserted into the pEF-DEST51 Gateway vector (Life Technologies Carlsbad, CA, USA)^7,18^. A Kozak cassette was introduced at the 5’ end before the start codon. For the surface expression assay of hTAS1R3, Myc tags with short flexible linker sequences were inserted in TAS1R3 construct after the signal peptide based on the sequence reported by the previous work. Myc-hATS1R3 was introduced into the pEF-DEST51 Gateway vector (Life Technologies Carlsbad, CA, USA). Human/mouse TAS1R chimeras were constructed by PCR using overlapping primers^60^. Point mutations of the TAS1R3 were made by site-directed mutagenesis (Takara Bio Inc., Shiga, Japan). DNA sequencing was performed to confirm the integrity of all the DNA constructs.

### Functional expression

HEK293 cells (provided by Dr. Makoto Tominaga (National Institutes of Natural Sciences, Japan)) were cultured in Dulbecco’s modified Eagle’s medium supplemented with 10% fetal bovine serum at 37 °C under a humidified atmosphere including 5% CO_2_. For cell surface staining and Ca^2+^ imaging, cells were seeded onto a 35-mm chamber (ibidi, Martinsried, Germany). After incubation for 24 h, the TAS1Rs and Gα16-gust44 (0.9 μg of each) were transiently cotransfected into HEK293 cells using Lipofectamine^TM^ 2000 transfection reagent (Thermo Fisher Scientific, Waltham, MA, USA; 2.5 μL per 0.9 μg DNA).

### Cell surface staining

Twenty-four hours after transfection, cells in 35-mm chambers were washed with phosphate-buffered saline (PBS) containing 15 mM NaN_3_ at room temperature^61^. The cells were reacted with anti-c-Myc–Alexa Fluor 647 (1:100; Cell Signaling; clone 9B11) antibodies in PBS containing 15 mM NaN_3_ at room temperature for 30 min. After washing with PBS containing 15 mM NaN_3_, the images of the positive Myc-hTAS1R3 cells were taken using the FV1000 confocal laser scanning microscope and Fluoview software (Olympus, Tokyo, Japan) and analyzed by ImageJ 1.47 T (National Institutes of health, USA).

### Single-cell Ca^2+^ imaging

Twenty-four hours after transfection, cells in 35-mm recording chambers were loaded with 3.0 mM fluo-4 acetoxymethyl ester (Thermo Fisher Scientific) for 30 min at 37 °C. After the cells had been washed with Hank’s balanced salt solution (HBSS; Thermo Fisher Scientific) containing 10 mM 4-(2-hydroxyethyl)-1-piperazineethanesulfonic acid (HEPES; pH 7.4), Ca^2+^ imaging was performed with a bath perfusion system to determine the activation kinetics. Taste solutions diluted in HBSS containing 10 mM HEPES were applied sequentially to the cells for 25 s with a peristaltic pump at a flow rate of 1.0 mL/min. Fluorescent images were captured with an S Fluor 620/0.75 objective lens (Nikon, Tokyo, Japan) via a cooled-CCD camera (C6790, Hamamatsu Photonics K.K., Shizuoka, Japan) fitted to a TE300 microscope (Nikon) or with a UplanXApo20x/0.80 objective lens (Olympus) via an sCMOS camera (Zyla, ANDOR, Tokyo, Japan) fitted to a IX73 microscope (Olympus). AquaCosmos 1.3 (Hamamatsu Photonics) or CellSens Dimension 4.1 (Olympus) was used to acquire and analyze the fluorescence images. A 5-min interval was maintained between the application of each tastant to ensure that the cells were not desensitized by the previous stimulation. Intracellular Ca^2+^ changes were measured from individual responding cells. Cells showing repeated increases in intracellular Ca^2+^ in the presence of sweet solution were counted as responding cells.

### Solutions

Solutions were diluted in HBSS containing 10 mM HEPES. The present study used saccharin (0.01, 0.03, 0.1, 0.3, 1, 3, 10 and 30 mM), SC45647 (0.3 mM), D-tryptophan (10 mM) and sodium cyclamate (0.1, 0.3, 1, 3, 10 and 30 mM) as sweet taste stimuli, lactisole (3 and 10 mM) as a sweet taste inhibitor, and isoproterenol (10 μM) as a positive control. The reagents were purchased from Ajinomoto Co., Inc., Tokyo, Japan (aspartame), MilliporeSigma, Burlington, MA, USA (cyclamate and lactisole), and Fujifilm Wako Pure Chemical Corporation, Osaka, Japan (others).

### Analysis of the functional expression system data

Intracellular Ca^2+^ changes in individual cells were monitored as changes in fluo-4 fluorescence. Fluorometric signals were expressed as relative fluorescence changes: ΔF/F_0_ = (F-F_0_)/F_0_, where F_0_ denotes the baseline fluorescence level. The magnitudes of the Ca^2+^ changes from 5 s to 25 s after stimulus onset were measured and averaged. The data are expressed as the mean ± S.E. of the ΔF/F_0_ value. Half-maximal effective concentration (EC_50_) values were calculated from individual concentration-response data using the curving-fitting routines of Origin 5.0 (OriginLab, Northampton, MA, USA).

### Statistics and reproducibility

The effects of genotype, pH and concentration on sweet responses were evaluated by one-way or two-way ANOVA and the post-hoc Tukey–Kramer test or *t*-test. SPSS Statistics software (IBM Corp., Armonk, NY, USA) was used for all calculations.

### Reporting summary

Further information on research design is available in the Nature Portfolio Reporting Summary linked to this article.

## Supplementary information

Supplementary Information

Description of Additional Supplementary Data

Supplementary Data 1

Supplementary Data 2

Supplementary Movie 1

Supplementary Movie 2

Supplementary Movie 3

Supplementary Movie 4

Reporting Summary

## Data Availability

The data are available from the corresponding author upon reasonable request. The initial and final PDB structure files of the full system and simulation input files can be obtained from Supplementary Data 1, and all source data underlying the graphs presented in the main figures are available as Supplementary Data 2.

## References

[1] Li X (2001). High-resolution genetic mapping of the saccharin preference locus (Sac) and the putative sweet taste receptor (T1R1) gene (Gpr70) to mouse distal Chromosome 4. Mamm. Genome.

[2] Nelson G (2001). Mammalian sweet taste receptors. Cell.

[3] Park J (2019). Structural architecture of a dimeric class C GPCR based on co-trafficking of sweet taste receptor subunits. J. Biol. Chem..

[4] Koehl A (2019). Structural insights into the activation of metabotropic glutamate receptors. Nature.

[5] Jiang P (2005). Lactisole interacts with the transmembrane domains of human T1R3 to inhibit sweet taste. J. Biol. Chem..

[6] Jiang P (2005). Identification of the cyclamate interaction site within the transmembrane domain of the human sweet taste receptor subunit T1R3. J. Biol. Chem..

[7] Sanematsu K (2014). Molecular mechanisms for sweet-suppressing effect of gymnemic acids. J. Biol. Chem..

[8] Winnig M, Bufe B, Kratochwil NA, Slack JP, Meyerhof W (2007). The binding site for neohesperidin dihydrochalcone at the human sweet taste receptor. BMC Struct. Biol..

[9] Masuda K (2012). Characterization of the modes of binding between human sweet taste receptor and low-molecular-weight sweet compounds. PLoS One.

[10] Galindo-Cuspinera V, Winnig M, Bufe B, Meyerhof W, Breslin PA (2006). A TAS1R receptor-based explanation of sweet ‘water-taste’. Nature.

[11] Chen, K. Y. M., Keri, D. & Barth, P. Computational design of G Protein-Coupled Receptor allosteric signal transductions. *Nat. Chem. Biol*. **16**, 77–86 (2020). 10.1038/s41589-019-0407-2 31792443

[12] Nuemket N (2017). Structural basis for perception of diverse chemical substances by T1r taste receptors. Nat. Commun..

[13] Maillet EL, Margolskee RF, Mosinger B (2009). Phenoxy herbicides and fibrates potently inhibit the human chemosensory receptor subunit T1R3. J. Med. Chem..

[14] Nakagita T (2020). Ibuprofen, a nonsteroidal anti-inflammatory drug, is a potent inhibitor of the human sweet taste receptor. Chem. Senses.

[15] Nakagita T (2019). Structural insights into the differences among lactisole derivatives in inhibitory mechanisms against the human sweet taste receptor. PLoS One.

[16] Kim S-K, Chen Y, Abrol R, Goddard WA, Guthrie B (2017). Activation mechanism of the G protein-coupled sweet receptor heterodimer with sweeteners and allosteric agonists. Proc. Natl. Acad. Sci. USA.

[17] Jang J, Kim SK, Guthrie B, Goddard WA (2021). Synergic effects in the activation of the sweet receptor GPCR heterodimer for various sweeteners predicted using molecular metadynamics simulations. J. Agric. Food Chem..

[18] Sanematsu K (2016). Intracellular acidification is required for full activation of the sweet taste receptor by miraculin. Sci. Rep..

[19] Wu H (2014). Structure of a class C GPCR metabotropic glutamate receptor 1 bound to an allosteric modulator. Science.

[20] Thal DM, Glukhova A, Sexton PM, Christopoulos A (2018). Structural insights into G-protein-coupled receptor allostery. Nature.

[21] García-Nafría J, Nehmé R, Edwards PC, Tate CG (2018). Cryo-EM structure of the serotonin 5-HT1B receptor coupled to heterotrimeric Go. Nature.

[22] Koehl A (2018). Structure of the μ-opioid receptor-Gi protein complex. Nature.

[23] Draper-Joyce CJ (2018). Structure of the adenosine-bound human adenosine A1 receptor-Gi complex. Nature.

[24] Zhou Q (2019). Common activation mechanism of class A GPCRs. Elife.

[25] Genheden S, Ryde U (2015). The MM/PBSA and MM/GBSA methods to estimate ligand-binding affinities. Expert Opin Drug Discov..

[26] Audet M, Bouvier M (2012). Restructuring G-protein- coupled receptor activation. Cell.

[27] Schönegge A (2017). Evolutionary action and structural basis of the allosteric switch controlling β2AR functional selectivity. Nat. Commun.

[28] Alhadeff R, Vorobyov I, Yoon HW, Warshel A (2018). Exploring the free-energy landscape of GPCR activation. Proc. Natl. Acad. Sci..

[29] Jacobson KA, Costanzi S, Paoletta S (2014). Computational studies to predict or explain G protein coupled receptor polypharmacology. Trends Pharmacol. Sci.

[30] Feng X, Ambia J, Chen KYM, Young M, Barth P (2017). Computational design of ligand-binding membrane receptors with high selectivity. Nat. Chem. Biol..

[31] Roth BL, Irwin JJ, Shoichet BK (2017). Discovery of new GPCR ligands to illuminate new biology. Nat. Chem. Biol.

[32] Yuan S, Filipek S, Palczewski K, Vogel H (2014). Activation of G-protein-coupled receptors correlates with the formation of a continuous internal water pathway. Nat. Commun..

[33] Seven AB (2021). G-protein activation by a metabotropic glutamate receptor. Nature.

[34] Gao Y (2021). Asymmetric activation of the calcium-sensing receptor homodimer. Nature.

[35] Kobilka BK (2007). G protein coupled receptor structure and activation. Biochim. Biophys. Acta.

[36] Kunishima N (2000). Structural basis of glutamate recognition by a dimeric metabotropic glutamate receptor. Nature.

[37] Shigemura N, Shirosaki S, Sanematsu K, Yoshida R, Ninomiya Y (2009). Genetic and molecular basis of individual differences in human umami taste perception. PLoS One.

[38] Xu H (2004). Different functional roles of T1R subunits in the heteromeric taste receptors. Proc. Natl. Acad. Sci. USA.

[39] Sainz E (2007). The G-protein coupling properties of the human sweet and amino acid taste receptors. Dev. Neurobiol..

[40] Ballesteros JA, Weinstein H (1995). Integrated methods for the construction of three-dimensional models and computational probing of structure-function relations in G-protein-coupled receptors. Methods Neurosci.

[41] Pin J-P, Galvez T, Prézeau L (2003). Evolution, structure, and activation mechanism of family 3/C G-protein-coupled receptors. Pharmacol. Ther.

[42] Zhang F (2010). Molecular mechanism of the sweet taste enhancers. Proc. Natl. Acad. Sci. USA.

[43] Sánchez R, Sali A (2000). Comparative protein structure modeling. Introduction and practical examples with modeller. Methods Mol. Biol.

[44] Muto T, Tsuchiya D, Morikawa K, Jingami H (2007). Structures of the extracellular regions of the group II/III metabotropic glutamate receptors. Proc. Natl. Acad. Sci. USA.

[45] Frisch, M. J. et al. *Gaussian 16, Revision A.03* (Gaussian, Inc., 2016).

[46] Morris GM (2009). AutoDock4 and AutoDockTools4: automated docking with selective receptor flexibility. J. Comput. Chem..

[47] *The PyMOL molecular graphics system, version 2.5.0a0 open-source* (Schrödinger, LLC, 2021).

[48] Jo S, Kim T, Iyer VG, Im W (2008). CHARMM-GUI: a web-based graphical user interface for CHARMM. J. Comput. Chem..

[49] Lee J (2020). CHARMM-GUI supports the Amber force fields. J. Chem. Phys..

[50] Wu EL (2014). CHARMM-GUI membrane builder toward realistic biological membrane simulations. J. Comput. Chem..

[51] Maier JA (2015). ff14SB: improving the accuracy of protein side chain and backbone parameters from ff99SB. J. Chem. Theory Comput..

[52] CJ D (2014). Lipid14: the Amber lipid force field. J. Chem. Theory Comput..

[53] Wang J, Wolf RM, Caldwell JW, Kollman PA, Case DA (2004). Development and testing of a general amber force field. J. Comput. Chem..

[54] Florová P, Sklenovský P, Banáš P, Otyepka M (2010). Explicit water models affect the specific solvation and dynamics of unfolded peptides while the conformational behavior and flexibility of folded peptides remain intact. J. Chem. Theory Comput..

[55] Case, D. A. et al. *AMBER 2018*, (University of California, San Francisco, 2018).

[56] Ryckaert J-P, Ciccotti G, Berendsen HJC (1977). Numerical integration of the cartesian equations of motion of a system with constraints: molecular dynamics of n-alkanes. J. Comput. Phys..

[57] York DM, Darden TA, Pedersen LG (1998). The effect of long‐range electrostatic interactions in simulations of macromolecular crystals: a comparison of the Ewald and truncated list methods. J. Chem. Phys..

[58] Grant BJ, Rodrigues APC, ElSawy KM, McCammon JA, Caves LSD (2006). Bio3d: an R package for the comparative analysis of protein structures. Bioinformatics.

[59] Mahalapbutr P (2019). Atomistic mechanisms underlying the activation of the G protein-coupled sweet receptor heterodimer by sugar alcohol recognition. Sci. Rep..

[60] Horton RM, Hunt HD, Ho SN, Pullen JK, Pease LR (1989). Engineering hybrid genes without the use of restriction enzymes: gene splicing by overlap extension. Gene.

[61] Shimizu M, Goto M, Kawai T, Yamashita A, Kusakabe Y (2014). Distinct human and mouse membrane trafficking systems for sweet taste receptors t1r2 and t1r3. PLoS One.

