# Supplementary material for: Prediction of dynamic allostery for the transmembrane domain of the sweet taste receptor subunit, TAS1R3
Source: Commun Biol. 2023 Apr 3;6:340. doi: 10.1038/s42003-023-04705-5 (PMC10070457; doi:10.1038/s42003-023-04705-5)
Supplement: Supplementary file 1 — Supplementary Information [file 42003_2023_4705_MOESM1_ESM.pdf]

Supplementary Information for:

Prediction of dynamic allostery for the transmembrane domain of the sweet taste receptor subunit, TAS1R3

Keisuke Sanematsu, Masato Yamamoto, Yuki Nagasato, Yuko Kawabata, Yu Watanabe, Shusuke Iwata, Shingo Takai, Kiyoshi Toko, Toshiro Matsui, Naohisa Wada, and Noriatsu Shigemura

The Supplementary Information contains

Supplementary Figures 1–20

Supplementary Tables 1–5

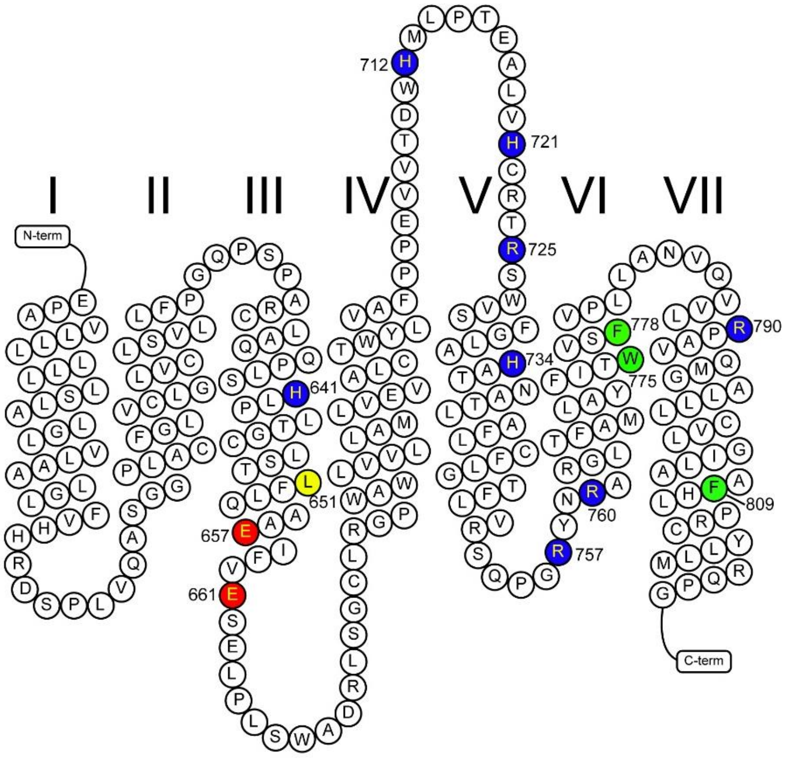

Supplementary Figure 1: Snake plot of the TMD of hTAS1R3.

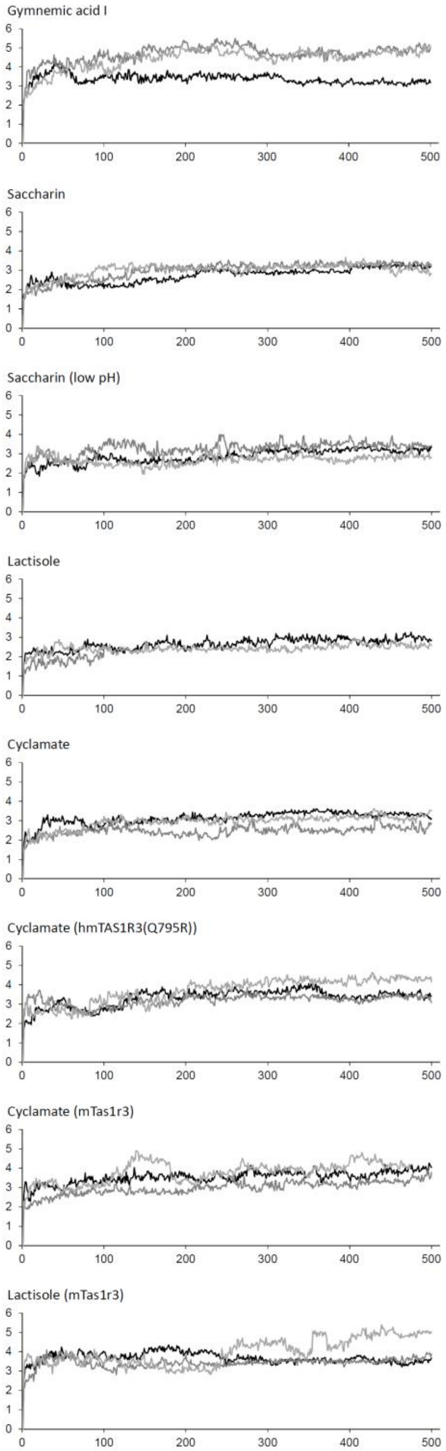

Supplementary Figure 2: RMSD of the Cα structure during MD simulations. RMSD of the Cα structure for hTAS1R3 TMD complexed with gymnemic acid I, saccharin (neutral and low pH model), lactisole, cyclamate, and, hmTAS1R3(Q795R) TMD complexed with cyclamate, and mTAS1R3 TMD complexed with lactisole and cyclamate (Trajectories used in the subsequent analysis are shown in black, n = 3).

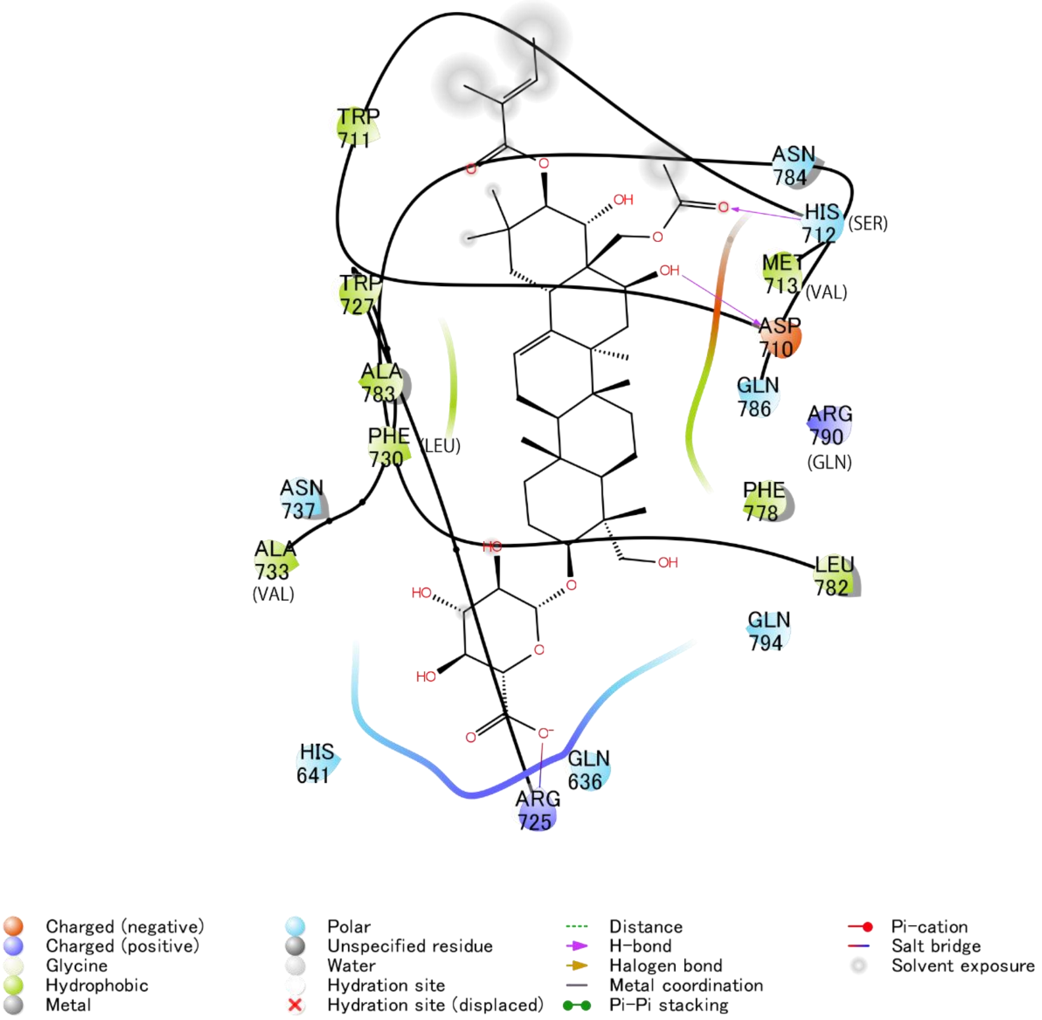

Supplementary Figure 3: Diagram of the interaction between gymnemic acid I and the hTAS1R3. The 2D interaction diagram shows the predicted binding of gymnemic acid I to the TMD of human TAS1R3 (500 ns). Mouse residues that are not conserved between human and mouse receptors are shown in parentheses.

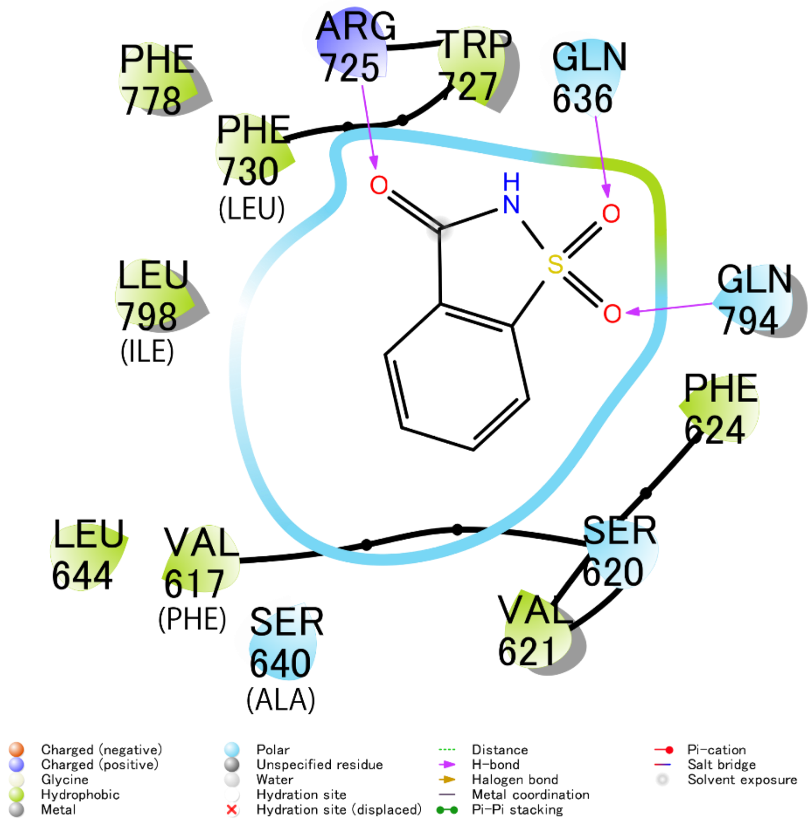

Supplementary Figure 4: Diagram of the interaction between saccharin and the hTAS1R3. The 2D interaction diagram shows the predicted binding of saccharin to the TMD of human TAS1R3 (500 ns). Mouse residues that are not conserved between human and mouse receptors are shown in parentheses.

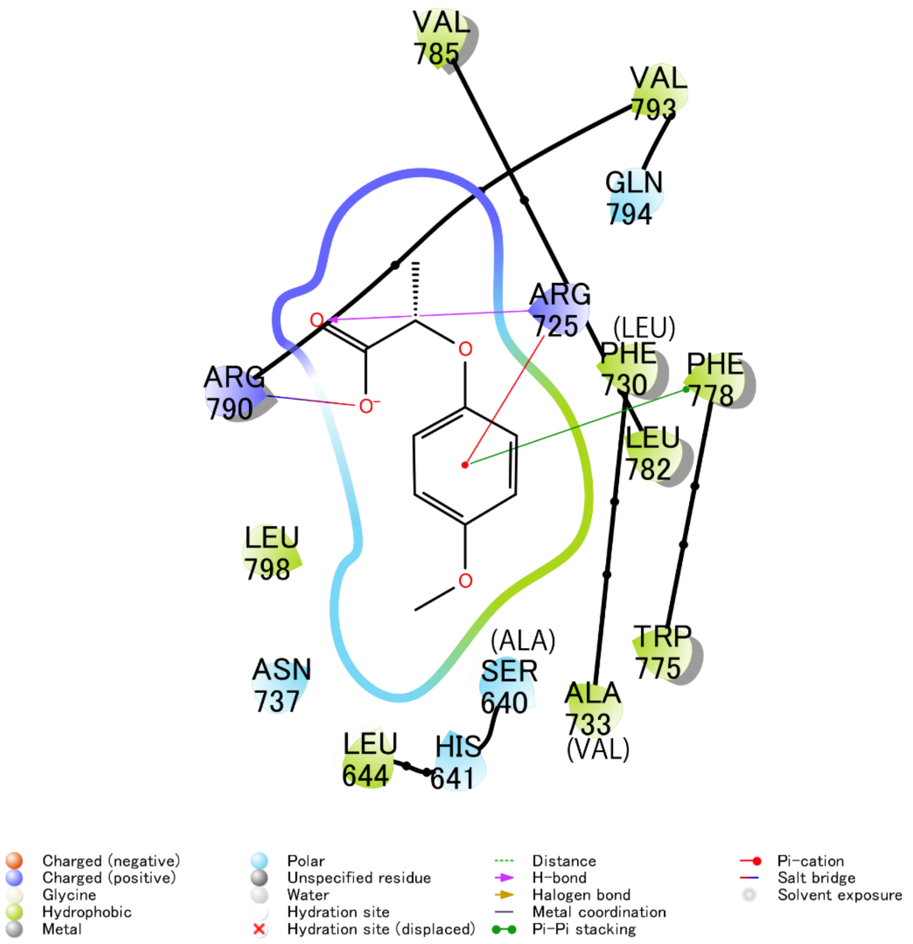

Supplementary Figure 5: Diagram of the interaction between lactisole and the hTAS1R3. The 2D interaction diagram shows the predicted binding of lactisole with the TMD of human TAS1R3 (500 ns). Mouse residues that are not conserved between human and mouse receptors are shown in parentheses.

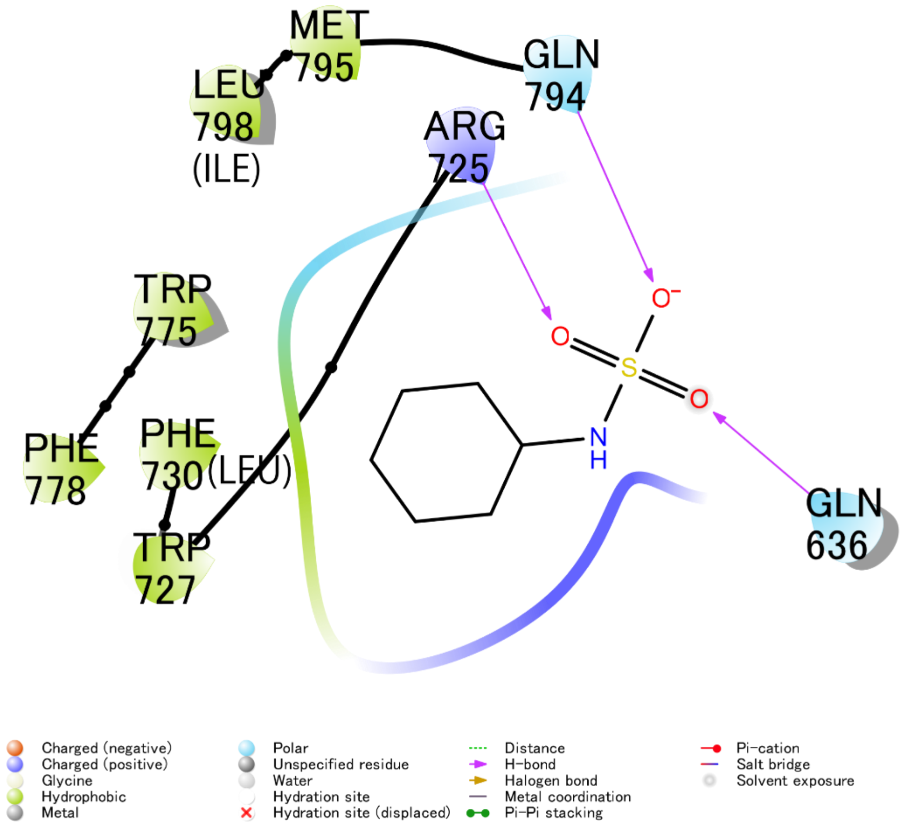

Supplementary Figure 6: Diagram of the interaction between cyclamate and the hTAS1R3. The 2D interaction diagram shows the predicted binding of cyclamate to the TMD of human TAS1R3 (500 ns). Mouse residues that are not conserved between human and mouse receptors are shown in parentheses.

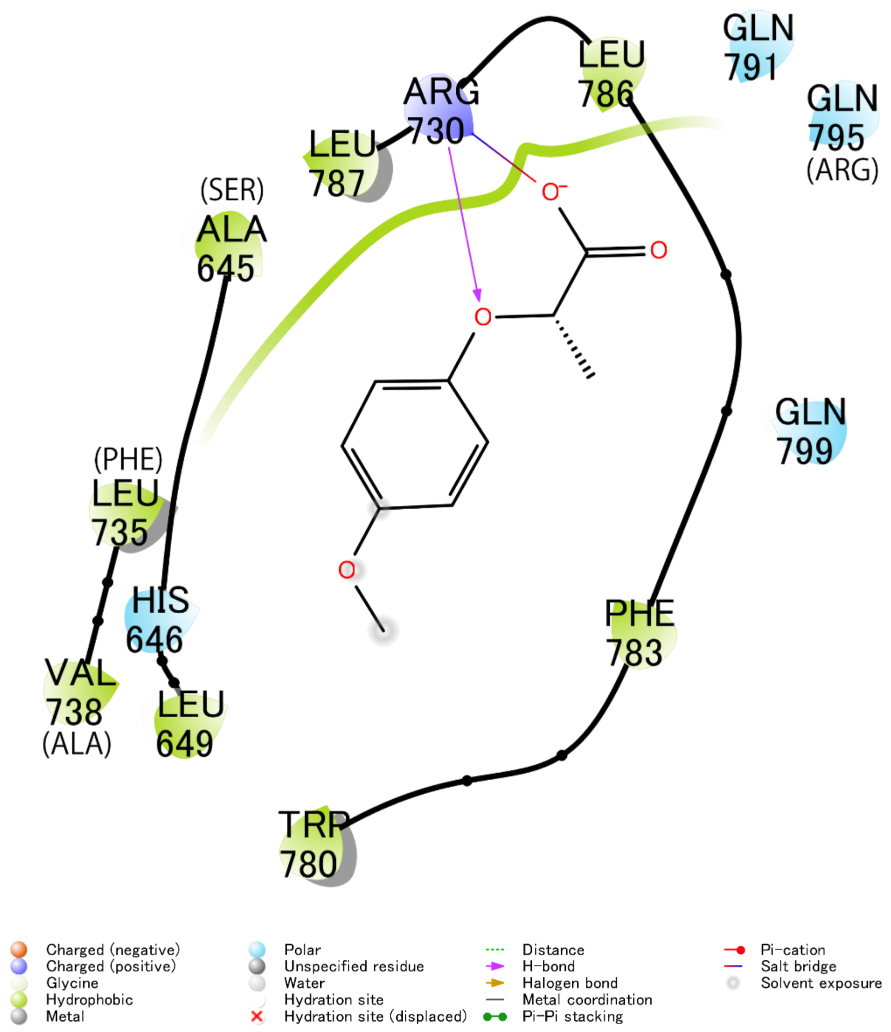

Supplementary Figure 7: Diagram of the interaction between lactisole and the mTas1r3. The 2D interaction diagram shows the predicted binding of lactisole to the TMD of mTas1r3 (500 ns). Human residues that are not conserved between human and mouse receptors are shown in parentheses.

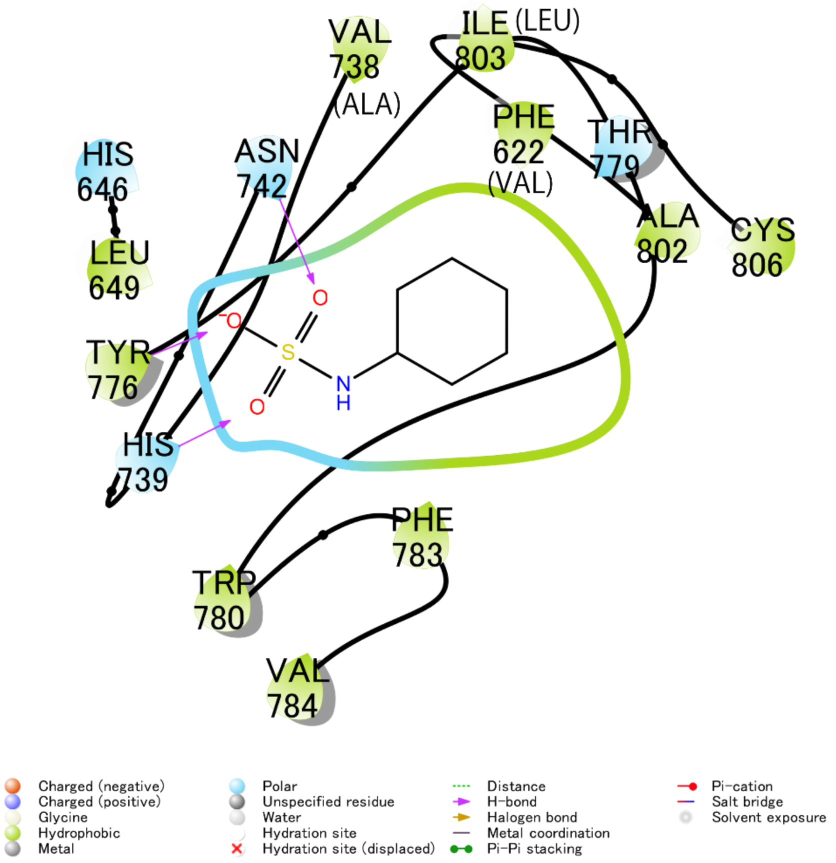

Supplementary Figure 8: Diagram of the interaction between cyclamate and the mTas1r3. The 2D interaction diagram shows the predicted binding of cyclamate to the TMD of mTas1r3 (500 ns). Human residues that are not conserved between human and mouse receptors are shown in parentheses.

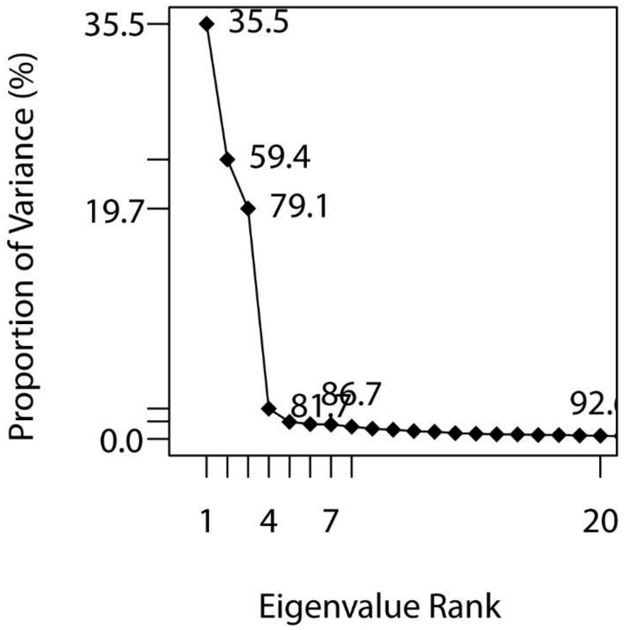

Supplementary Figure 9: PCA scree plots. Conformations of the hTAS1R3 Cα geometry in complex with cyclamate, saccharin, gymnemic acid I and lactisole throughout the MD trajectories (last 100 ns) were used for PCA. The proportion of variance is shown. The figure is based on data shown in Fig. 2b.

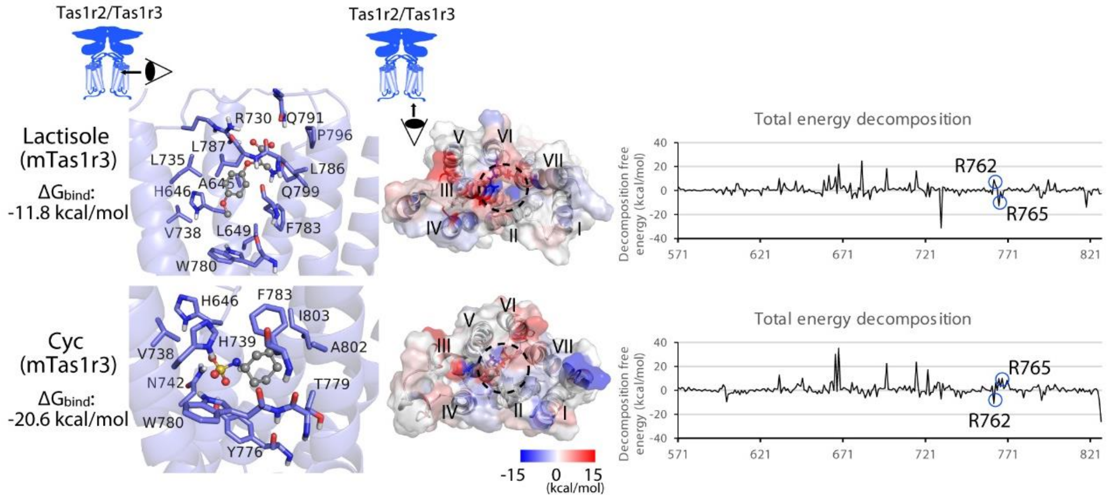

Supplementary Figure 10: Lactisole and cyclamate weakly bind to the mTas1r3 TMD. Left, detailed views of the mTas1r3 TMD binding pocket complexed with lactisole or cyclamate (Cyc) after 500 ns of MD simulation. The binding free energies (ΔG_{bind}) determined by the MM/PBSA method are indicated. Middle, molecular surface of the intracellular region of the mTas1r3 TMD colored according to decomposition energy. Dotted line indicates the interface with the Gα subunit. Right, total energy decomposition for each residue of the mTas1r3.

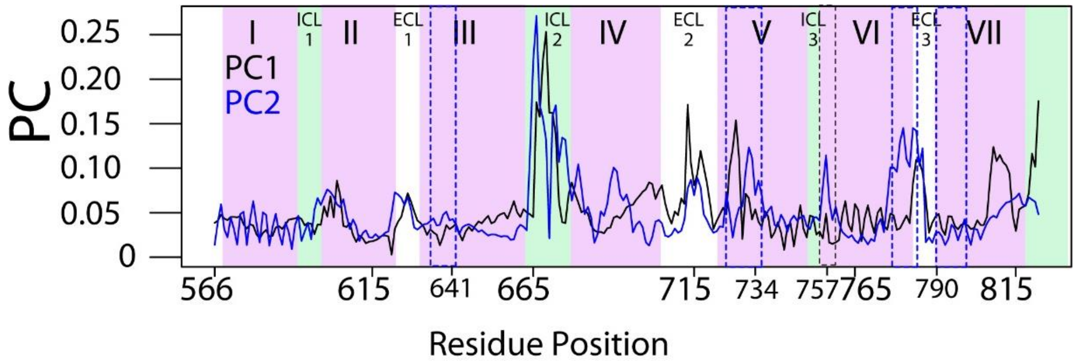

Supplementary Figure 11: Contribution of each residue to the first two principal components of PCA. The figure is based on data shown in Fig. 2b and Supplementary Fig. 9.

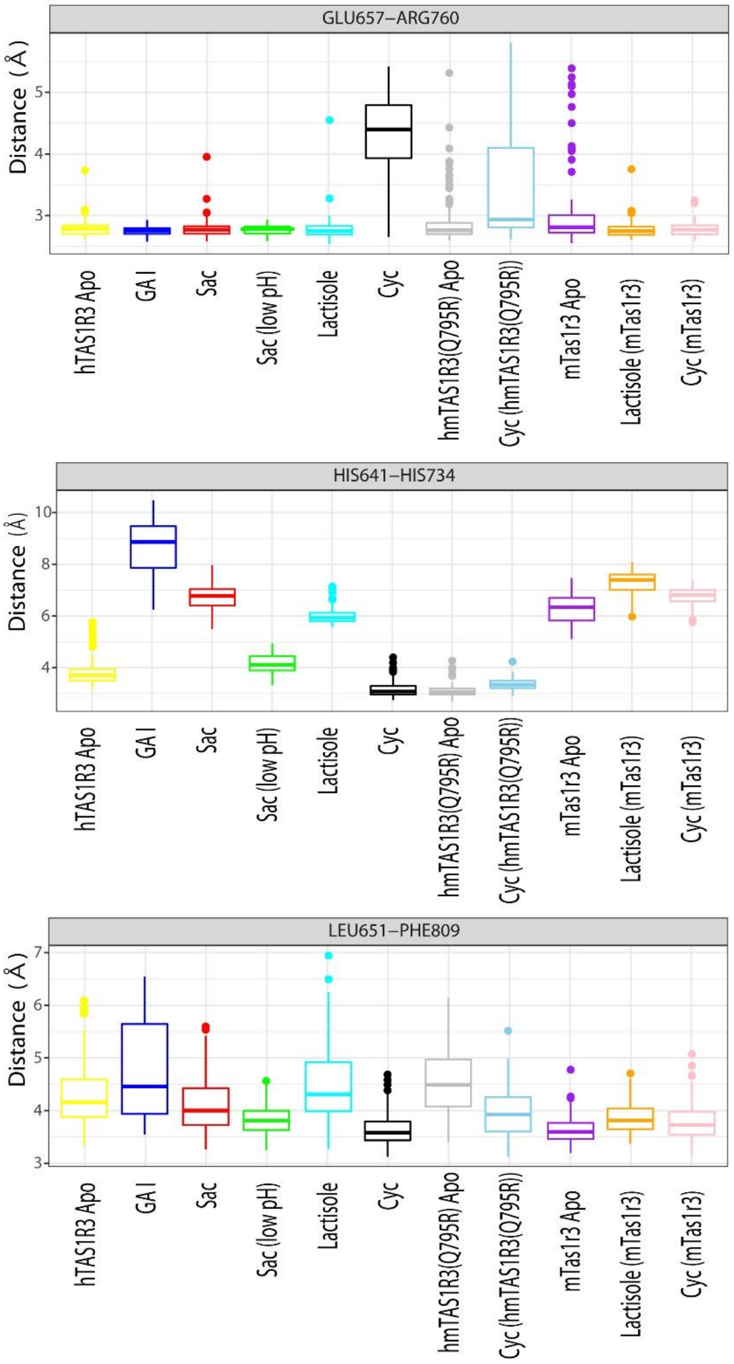

Supplementary Figure 12: Box-whisker plots of the distances between selected residue pairs. Distances between selected residue pairs for the last 100-ns structures are shown for the apo form of hTAS1R3 TMD, hTAS1R3 TMD in complex with gymnemic acid I (GA I), saccharin (Sac), lactisole and cyclamate (Cyc), protonated hTAS1R3 TMD in complex with Sac (low pH), apo form of the hmTAS1R3(Q795R) TMD, hmTAS1R3(Q795R) TMD in complex with Cyc and apo form of the mTAS1R3, mTAS1R3 TMD in complex with lactisole and Cyc. Equivalent human residue numbers are used for the mouse residue numbers.

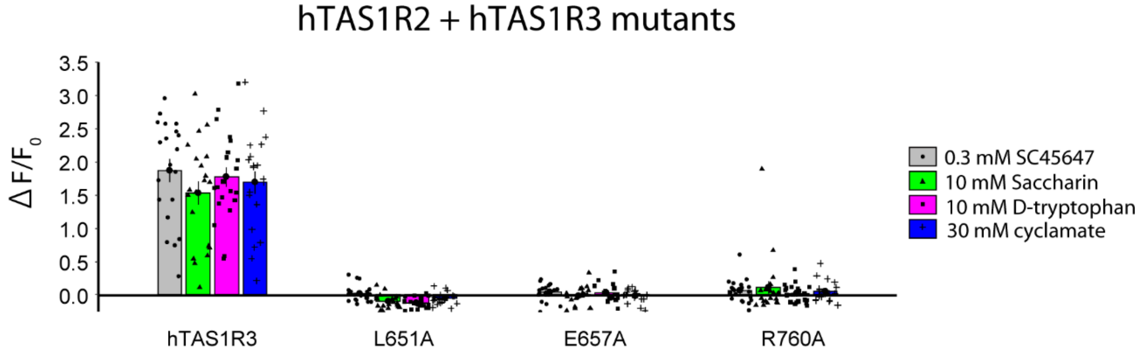

Supplementary Figure 13: Mutation of hTAS1R3 attenuates the sensitivity to sweet compounds. HEK293 cells were transiently transfected with TAS1R3 or the indicated hTAS1R3 mutants together with TAS1R2 and Gα16-gust44. The responses of the receptors to 0.3 mM SC45647, 10 mM saccharin, 10 mM D-tryptophan, and 30 mM cyclamate were examined. Data are expressed as the mean ± S.E. of 21 cells (n = 3).

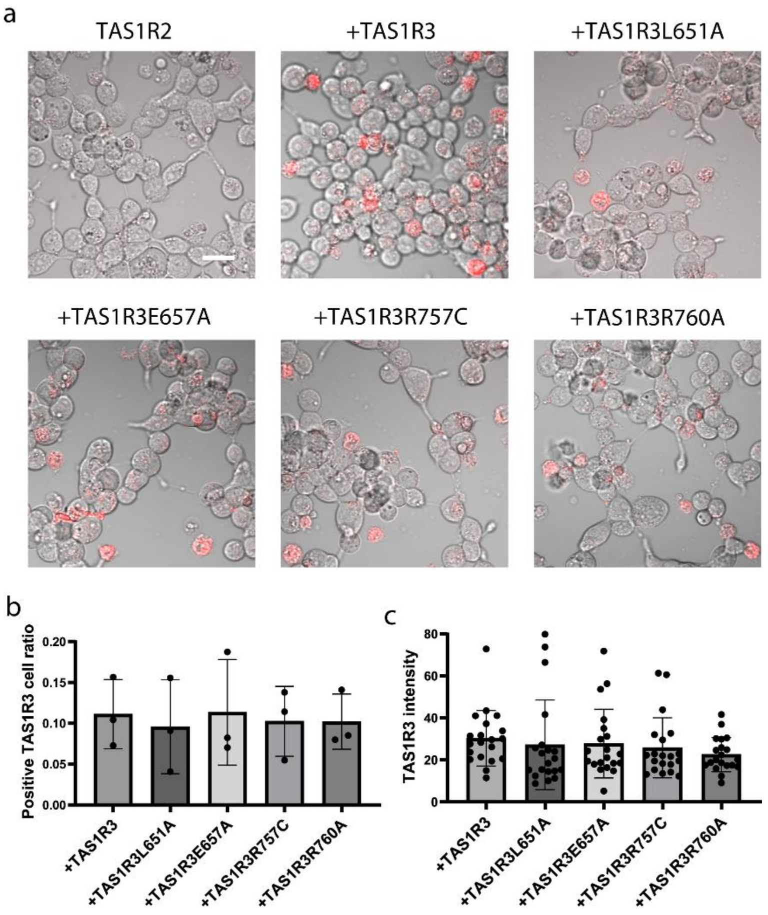

Supplementary Figure 14: Surface expression of TAS1R3 and TAS1R3 mutants. HEK293 cells were transiently co-transfected with hTAS1R2 alone, hTAS1R2+hTAS1R3, and hTAS1R2+the indicated hTAS1R3 mutants, along with Ga16-gust44. (**a**) Surface expression of the hTAS1R3 (anti-c-Myc–Alexa 647) was detected by confocal immunofluorescence microscopy of nonpermeabilized cells. No immunoreactivity was detected in HEK293 cells expressing hTAS1R2 alone. Scale bar = 20 µm. (**b**) The number of positive hTAS1R3 (anti-c-Myc–Alexa 647) cells was counted and the ratio (positive hTAS1R3 cells / all cells) is presented. Data are expressed as the mean ± S.E. (n = 3 from 160-321 cells). There was no significant difference between TAS1R3 and TAS1R3 mutants (one-way ANOVA). (**c**) Graph is TAS1R3 fluorescence intensity of cell surface. Data are expressed as the mean ± S.E of 20 cells (n = 3). There was no significant difference between TAS1R3 and TAS1R3 mutants (one-way ANOVA).

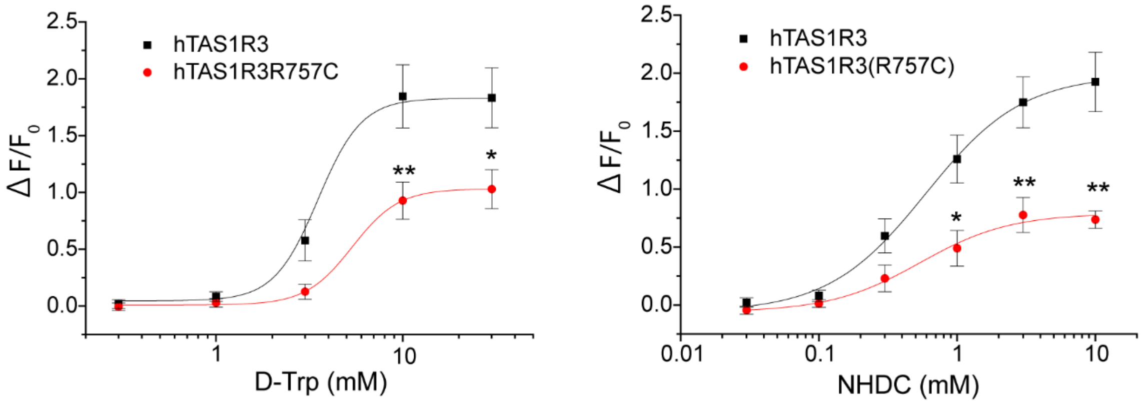

Supplementary Figure 15: Mutation of hTAS1R3 (R757C) reduces the sensitivity to sweet compounds. HEK293 cells were transiently transfected with hTAS1R3 or hTAS1R3(R757C) along with hTAS1R2 and Gα16-gust44. The responses of the receptors to various concentrations of D-tryptophan and neohesperidin dihydrochalcone (NHDC) were examined. Data are expressed as the mean ± S.E. of 14–19 cells (n = 3). *$P < 0.05$, **$P < 0.01$ for the comparison between hTAS1R3 and hTAS1R3(R757C) (two-way ANOVA and post-hoc t-test).

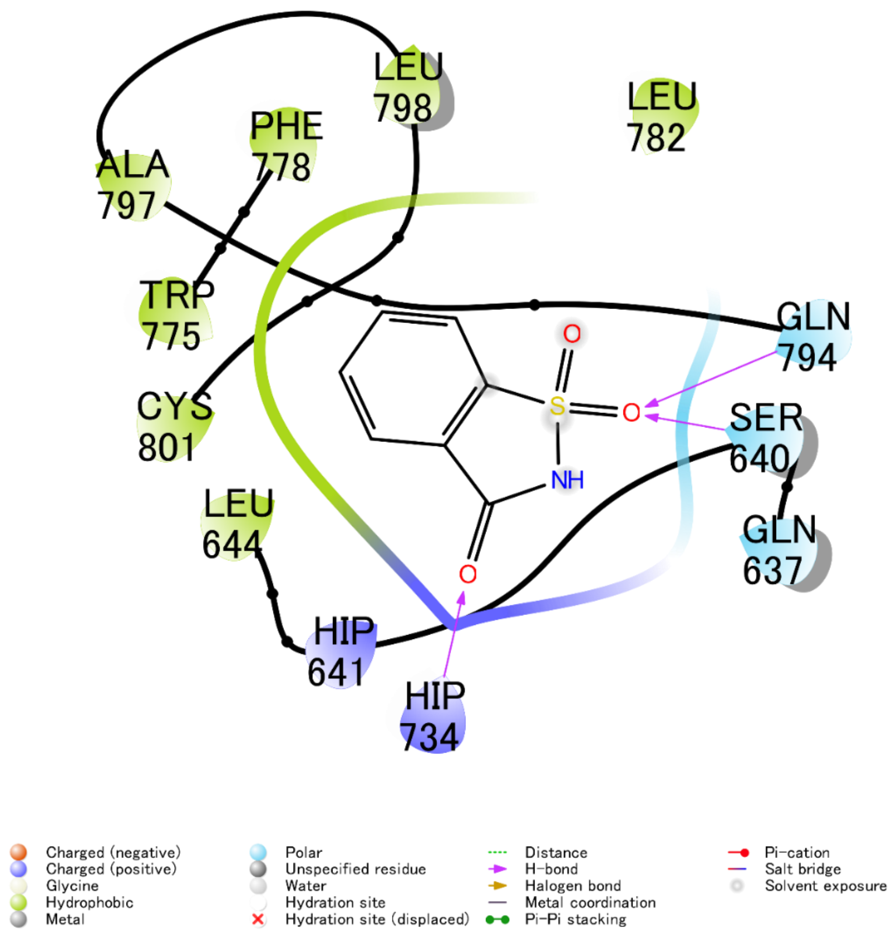

Supplementary Figure 16: Diagram of the interaction between saccharin and the protonated hTAS1R3. The 2D interaction diagram shows the predicted binding of saccharin to the TMD of the human TAS1R3 (500 ns). His-641, -712, -721 and -734 of the hTAS1R3 were protonated and are shown as HIP.

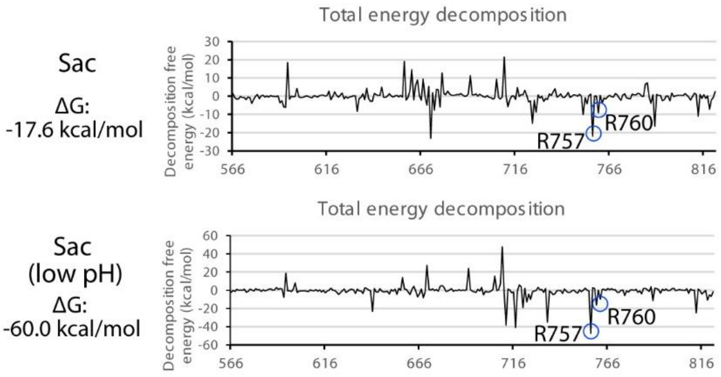

Supplementary Figure 17: Total energy decomposition of the hTAS1R3 TMD complexed with saccharin under neutral and low pH. Gibbs free energies (ΔG) are indicated. Total energy decomposition for each residue was performed for neutral and protonated hTAS1R3 TMD in complex with saccharin.

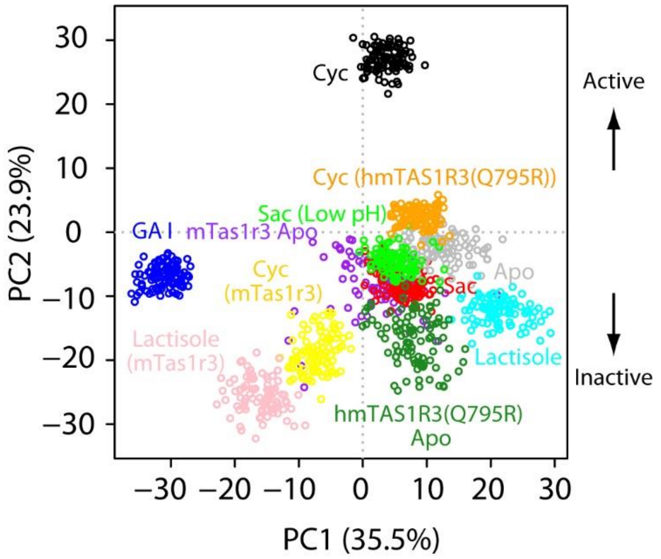

Supplementary Figure 18: PCA of trajectory frames with instantaneous conformations. Structures (last 100 ns) of protonated hTAS1R3 TMD complexed with saccharin (green) were projected onto the axis of Fig. 2b. Each point represents a structure. hTAS1R3 TMD complexed with cyclamate (Cyc, black) and hmTAS1R3(Q795R) TMD complexed with Cyc (orange) represent active GPCR structures. hTAS1R3 TMD complexed with GAI (blue), Sac (red) and lactisole (cyan) represent inactive GPCR structures. mTAS1R3 TMD complexed with lactisole and Cyc and the apo forms of the hTAS1R3 TMD, hmTAS1R3(Q795R) and mTAS1R3 TMD are shown in pink, yellow, gray, forest green and purple, respectively.

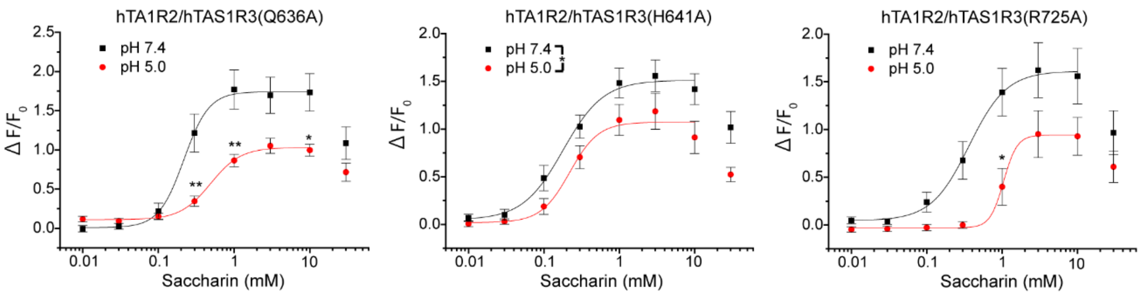

Supplementary Figure19: Mutations of hTAS1R3 (Q636A, H641A, and R725A) do not affect the pH-dependence of the sensitivity to saccharin. HEK293 cells were transiently transfected with TAS1R3 or TAS1R3(Q636A, H641A or R725A) together with TAS1R2 and Gα16-gust44. The responses of the receptors to different concentrations of saccharin were examined at neutral pH (7.4) and low pH (5.0). Data are expressed as the mean ± S.E. of 13–20 cells ($n = 3$). *$P < 0.05$, **$P < 0.01$ for the comparison between neutral pH and low pH (two-way ANOVA and post-hoc t-test).

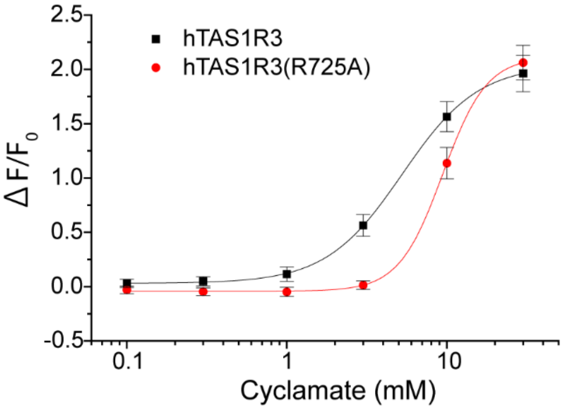

Supplementary Figure 20: Concentration-response curve to cyclamate in HEK293 cells expressing hTAS1R2/hTAS1R3 and hTAS1R2/hTAS1R3(R725A). HEK293 cells were transiently transfected with hTAS1R3 or hTAS1R3(R725A) along with hTAS1R2 and Gα16-gust44. The responses of the receptors to various concentrations of cyclamate were examined. Data are expressed as the mean ± S.E. of 19–20 cells (n = 3). ($P > 0.05$, the effect of genotype, two-way ANOVA).

Supplementary Table 1: Effects of cyclamate and lactisole on the calcium responses to sweet substances. The effects of cyclamate and lactisole on the responses to various sweet substances (0.3 mM SC45647, 10 mM saccharin or 10 mM D-tryptophan) were analyzed by one-way ANOVA. The table is based on data shown in Fig. 2c, d. *$P < 0.05$, **$P < 0.01$, ***$P < 0.001$.

	Cyclamate		Lactisole	
	Degrees of freedom	*F* value	Degrees of freedom	*F* value
SC45647	2, 57	26.71***	2, 57	3.68*
Saccharin	2, 57	14.41***	2, 57	5.92**
D-tryptophan	2, 57	11.14***	2, 57	4.06*

Supplementary Table 2: Effects of genotype on the surface expression in HEK293 cells. The effects of genotypes on the ratio and the intensity of positive hTAS1R3 cells were analyzed by one-way ANOVA. The table is based on data shown in Supplementary Fig. 14. $*P < 0.05$, $**P < 0.01$, $***P < 0.001$.

	Ratio		Intensity	
	Degrees of freedom	*F* value	Degrees of freedom	*F* value
Genotypes	4, 10	0.063	4, 95	0.70

Supplementary Table 3: Comparison of the calcium responses to sweet compounds between hTAS1R2/hTAS1R3 and hTAS1R2/hTAS1R3(R757C). The effect of genotype was analyzed by two-way ANOVA. The table is based on data shown in Fig. 4d and Supplementary Fig. 15. $^{**}P < 0.01$, $^{***}P < 0.001$.

	Genotype		Genotype × Concentration	
	Degrees of freedom	*F* value	Degrees of freedom	*F* value
Cyclamate	1, 165	19.05**	5, 165	21.62***
D-tryptophan	1, 128	7.35*	4, 128	6.28***
NHDC	1, 155	10.07**	5, 155	10.79***

Supplementary Table 4: Effects of pH on the calcium responses to saccharin in hTAS1R2/hTAS1R3, mTas1r2/mTas1r3, mTas1r2/mhTAS1R3, hTAS1R2/hmTAS1R3, hTAS1R2/hTAS1R3(Q636A), hTAS1R2/hTAS1R3(H641A), hTAS1R2/hTAS1R3(R725A), and hTAS1R2/hTAS1R3(H641AR725A). The effect of pH (pH 7.4 vs. pH 5.0) was analyzed by two-way ANOVA. The table is based on data shown in Fig. 5c–f, i and Supplementary Fig. 19. $^{*}P < 0.05$, $^{**}P < 0.01$, $^{***}P < 0.001$.

	pH		pH × Concentration	
	Degrees of freedom	*F* value	Degrees of freedom	*F* value
hTAS1R2/hTAS1R3	1, 294	10.33**	7, 294	5.11***
mTas1r2/mTas1r3	1, 266	0.074	7, 266	2.51
mTas1r2/mhTAS1R3	1, 294	27.86***	7, 294	39.32***
hTAS1R2/hmTAS1R3	1, 217	0.0043	7, 217	3.41**
hTAS1R2/hTAS1R3(Q636A)	1, 238	8.33**	7, 238	7.96***
hTAS1R2/hTAS1R3(H641A)	1, 266	5.07*	7, 266	1.88
hTAS1R2/hTAS1R3(R725A)	1, 168	7.45*	7, 168	2.75**
hTAS1R2/hTAS1R3(H641AR725A)	1, 236	2.50	7, 236	0.60

Supplementary Table 5: Comparison of the calcium responses to sweet compounds between hTAS1R2/hTAS1R3 and hTAS1R2/hTAS1R3(R725A). The effect of genotype was analyzed by two-way ANOVA. The table is based on data shown in Supplementary Fig. 20. ***$P < 0.001$.

	Genotype		Genotype × Concentration	
	Degrees of freedom	*F* value	Degrees of freedom	*F* value
Cyclamate	1, 185	3.99	5, 185	5.22***
