# Supplementary material for: Prediction of dynamic allostery for the transmembrane domain of the sweet taste receptor subunit, TAS1R3
Source: Commun Biol. 2023 Apr 3;6:340. doi: 10.1038/s42003-023-04705-5 (PMC10070457; doi:10.1038/s42003-023-04705-5)
Supplement: Supplementary file 2 — Description of Additional Supplementary Data [file 42003_2023_4705_MOESM2_ESM.docx]

**Description of Additional Supplementary Files**

**File name:** Supplementary Data 1

**Description:** The initial and final PDB structure files of the full system and simulation input files

**File name:** Supplementary Data 2

**Description:** The source data behind the graphs in the paper

**File name:** Supplementary Movie 1

**Description:** Molecular dynamics simulation for the transmembrane domain (TMD) of the human taste type 1 receptor member 3 (hTAS1R3) bound with gymnemic acid I. The hTAS1R3 TMD and gymnemic acid I are represented as ribbons and spheres colored by atom type, respectively.

**File name:** Supplementary Movie 2

**Description:** Molecular dynamics simulation for the transmembrane domain (TMD) of the mouse taste type 1 receptor member 3 (mTas1r3) bound with gymnemic acid I. The mTas1r3 TMD and gymnemic acid I are represented as ribbons and spheres colored by atom type, respectively.

**File name:** Supplementary Movie 3

**Description:** Lactisole stabilizes the formation of an ionic lock in the transmembrane domain (TMD) of the human taste type 1 receptor member 3 (hTAS1R3). The hTAS1R3 TMD, key residues and lactisole are represented as ribbons, sticks and spheres colored by atom type, respectively. Yellow dotted lines indicate hydrogen bonds.

**File name:** Supplementary Movie 4

**Description:** Cyclamate induces ionic lock opening in the transmembrane domain (TMD) of the human taste type 1 receptor member 3 (hTAS1R3). The hTAS1R3 TMD, key residues and cyclamate are represented as ribbons, sticks and spheres colored by atom type, respectively. Yellow dotted lines indicate hydrogen bonds.
