# Supplementary material for: Prediction of dynamic allostery for the transmembrane domain of the sweet taste receptor subunit, TAS1R3
Source: Commun Biol. 2023 Apr 3;6:340. doi: 10.1038/s42003-023-04705-5 (PMC10070457; doi:10.1038/s42003-023-04705-5)
Supplement: Supplementary file 9 — Reporting Summary [file 42003_2023_4705_MOESM9_ESM.pdf]

nature portfolio

Corresponding author(s): Keisuke Sanematsu, Noriatsu Shigemura

Last updated by author(s): Mar 10, 2023

Reporting Summary

Nature Portfolio wishes to improve the reproducibility of the work that we publish. This form provides structure for consistency and transparency in reporting. For further information on Nature Portfolio policies, see our Editorial Policies and the Editorial Policy Checklist.

Statistics

For all statistical analyses, confirm that the following items are present in the figure legend, table legend, main text, or Methods section.

n/a	Confirmed	
☐	☒	The exact sample size (*n*) for each experimental group/condition, given as a discrete number and unit of measurement
☐	☒	A statement on whether measurements were taken from distinct samples or whether the same sample was measured repeatedly
☐	☒	The statistical test(s) used AND whether they are one- or two-sided<br>*Only common tests should be described solely by name; describe more complex techniques in the Methods section.*
☐	☒	A description of all covariates tested
☐	☒	A description of any assumptions or corrections, such as tests of normality and adjustment for multiple comparisons
☐	☒	A full description of the statistical parameters including central tendency (e.g. means) or other basic estimates (e.g. regression coefficient) AND variation (e.g. standard deviation) or associated estimates of uncertainty (e.g. confidence intervals)
☐	☒	For null hypothesis testing, the test statistic (e.g. *F*, *t*, *r*) with confidence intervals, effect sizes, degrees of freedom and *P* value noted<br>*Give P values as exact values whenever suitable.*
☒	☐	For Bayesian analysis, information on the choice of priors and Markov chain Monte Carlo settings
☒	☐	For hierarchical and complex designs, identification of the appropriate level for tests and full reporting of outcomes
☒	☐	Estimates of effect sizes (e.g. Cohen's *d*, Pearson's *r*), indicating how they were calculated

Our web collection on statistics for biologists contains articles on many of the points above.

Software and code

Policy information about availability of computer code

Data collection	AquaCosmos software (v. 1.3), CellSens Dimension 4.1, Fluoview, Modeller9.20, Gaussian16 A.03, AutoDock4, AMBER18
Data analysis	GaussView_6.1.1, AutoDockTools-1.5.6, Bio3d_2.4, UCSF Chimera_1.14, Maestro 12.6, Pymol2.5.0a0, Excel2019, Origin50, DaVinci Resolve 17, ImageJ 1.47t

For manuscripts utilizing custom algorithms or software that are central to the research but not yet described in published literature, software must be made available to editors and reviewers. We strongly encourage code deposition in a community repository (e.g. GitHub). See the Nature Portfolio guidelines for submitting code & software for further information.

Data

Policy information about availability of data

All manuscripts must include a data availability statement. This statement should provide the following information, where applicable:

- Accession codes, unique identifiers, or web links for publicly available datasets
- A description of any restrictions on data availability
- For clinical datasets or third party data, please ensure that the statement adheres to our policy

The data that support the findings of this study are available from the corresponding authors upon reasonable request.

Human research participants

Policy information about [studies involving human research participants and Sex and Gender in Research.]

Reporting on sex and gender	N/A
Population characteristics	N/A
Recruitment	N/A
Ethics oversight	N/A

Note that full information on the approval of the study protocol must also be provided in the manuscript.

Field-specific reporting

Please select the one below that is the best fit for your research. If you are not sure, read the appropriate sections before making your selection.

☒ Life sciences ☐ Behavioural & social sciences ☐ Ecological, evolutionary & environmental sciences

For a reference copy of the document with all sections, see nature.com/documents/nr-reporting-summary-flat.pdf

Life sciences study design

All studies must disclose on these points even when the disclosure is negative.

Sample size	No sample size calculations were made because this is not a population study.
Data exclusions	No data were excluded from analysis
Replication	All biological experiments were independently reproduced for three times.
Randomization	Randomization of groups/samples is not relevant to our study because no animal or human subjects are involved
Blinding	For functional analysis, blinding was not necessary by reason of the quantitative nature of the experiment. No human judgment was involved in generating the data and hence blinding is not required for this study.

Reporting for specific materials, systems and methods

We require information from authors about some types of materials, experimental systems and methods used in many studies. Here, indicate whether each material, system or method listed is relevant to your study. If you are not sure if a list item applies to your research, read the appropriate section before selecting a response.

Materials & experimental systems

n/a	Involved in the study	
☐	☒	Antibodies
☐	☒	Eukaryotic cell lines
☒	☐	Palaeontology and archaeology
☒	☐	Animals and other organisms
☒	☐	Clinical data
☒	☐	Dual use research of concern

Methods

n/a	Involved in the study	
☒	☐	ChIP-seq
☒	☐	Flow cytometry
☒	☐	MRI-based neuroimaging

Antibodies

Antibodies used	Myc-Tag (9B11) Mouse mAb (Alexa Fluor® 647 Conjugate) (Cell Signaling; clone 9B11)
Validation	https://www.cellsignal.jp/products/antibody-conjugates/myc-tag-9b11-mouse-mab-alexa-fluor-647-conjugate/2233

Eukaryotic cell lines

Policy information about cell lines and Sex and Gender in Research

Cell line source(s)	HEK293 from Dr. Makoto Tominaga at National Institutes of Natural Sciences, Okazaki, Japan
Authentication	No further authentications were performed for this study.
Mycoplasma contamination	No mycoplasma contamination tests were performed for this study.
Commonly misidentified lines (See ICLAC register)	No commonly misidentified cell lines were used in this study.
